# Leveraging uncertainty estimates for drug response prediction in cancer cell lines

**DOI:** 10.1093/bioadv/vbag246

**Published:** 2026-08-25

**Authors:** Pascal Iversen, Bernhard Y Renard, Katharina Baum

**Affiliations:** Department of Mathematics and Computer Science, Freie Universität Berlin, Berlin, Germany; Hasso Plattner Institute, Digital Engineering Faculty, University of Potsdam, Potsdam, Germany; Hasso Plattner Institute, Digital Engineering Faculty, University of Potsdam, Potsdam, Germany; Windreich Department of Artificial Intelligence and Human Health & Hasso Plattner Institute for Digital Health at Mount Sinai, Icahn School of Medicine at Mount Sinai, New York, NY, United States; Department of Mathematics and Computer Science, Freie Universität Berlin, Berlin, Germany; Hasso Plattner Institute, Digital Engineering Faculty, University of Potsdam, Potsdam, Germany; Windreich Department of Artificial Intelligence and Human Health & Hasso Plattner Institute for Digital Health at Mount Sinai, Icahn School of Medicine at Mount Sinai, New York, NY, United States

## Abstract

**Motivation:**

Machine learning models for drug response prediction in cancer cell lines could advance precision oncology by tailoring treatments to the molecular tumor profile. Their application is challenged by variability in prediction quality and distribution shifts between training and application. Yet, the most effective uncertainty estimator is domain-specific. We benchmark uncertainty-aware models for drug response prediction. We focus on epistemic uncertainty via ensemble agreement, and aleatoric uncertainty via distributional modeling, or both.

**Results:**

We find that ensemble-based estimates are more sensitive to distribution shift and can flag out-of-distribution examples. In contrast, distributional models yield stronger prediction error reductions among high-confidence subsets. The combination can provide both advantages: an ensemble of neural networks that estimate a Gaussian predictive distribution can reduce the mean squared error by 64% when restricting predictions to the 10% most confident drug-cell line pairs, and reliably indicates distribution shifts and platform differences. We show that uncertainty estimates enable a new dimension of model interpretability: by attributing predicted uncertainty to input features, we identify genes that signal unpredictability of drug response rather than sensitivity or resistance. We further demonstrate uncertainty-guided selection of measurements for active learning.

**Availability and implementation:**

The code is available at https://github.com/PascalIversen/LUDRP.

## 1 Introduction

Data-driven drug response prediction models aim to identify drug-specific sensitivity or resistance patterns in the omics profile of tumor cell lines (Barretina *et al*. 2012). These models carry the potential to improve therapeutic outcomes by informing preclinical studies or ultimately medical decisions. Large-scale projects such as Genomics of Drug Sensitivity in Cancer (GDSC) screened tumor-derived, cultured cell lines against a wide array of anti-cancer agents (Yang *et al*. 2013), enabling Machine Learning (ML)-based preclinical drug response prediction models. These models can be fine-tuned on data that better represent clinical tumor cases, such as xenografts, and used to identify therapeutic markers using explainable ML, or to recommend drug repurposing candidates (Lee *et al*. 2017, Manica *et al*. 2019, Prasse *et al*. 2022). Despite abundant research, challenges persist that hinder their application: Our data-leakage-free, unbiased evaluations show that population-wide model errors are high (Bernett *et al*., 2026). In practice, the prediction error is heteroscedastic, i.e. the prediction quality varies considerably between cell line-drug pairs. Therefore, a naive empirical risk estimate on the test set is a weak estimate for the model’s prediction performance on a specific cell line-drug pair. Further, even if drug response models can be improved to achieve a high average accuracy across the population, they can still show substantial errors for specific cell-line drug pairs (Chakraborti *et al*. 2025).

Additionally, distribution shift (systematic differences between the training and application data) can occur when transferring across screening platforms or to more complex preclinical systems (Haibe-Kains *et al*. 2013). This can lead to a silent failure of applied drug response prediction models. The cell line panels may not adequately represent the diversity of the target application, or measurement conditions might change inadvertently (Raghavan 2022). Such shifts have caused failures in applied biomedical ML (Zech *et al*. 2018, Obermeyer *et al*. 2019, Wong *et al*. 2021), which motivates us to characterize model robustness early in development.

**Figure 1:**
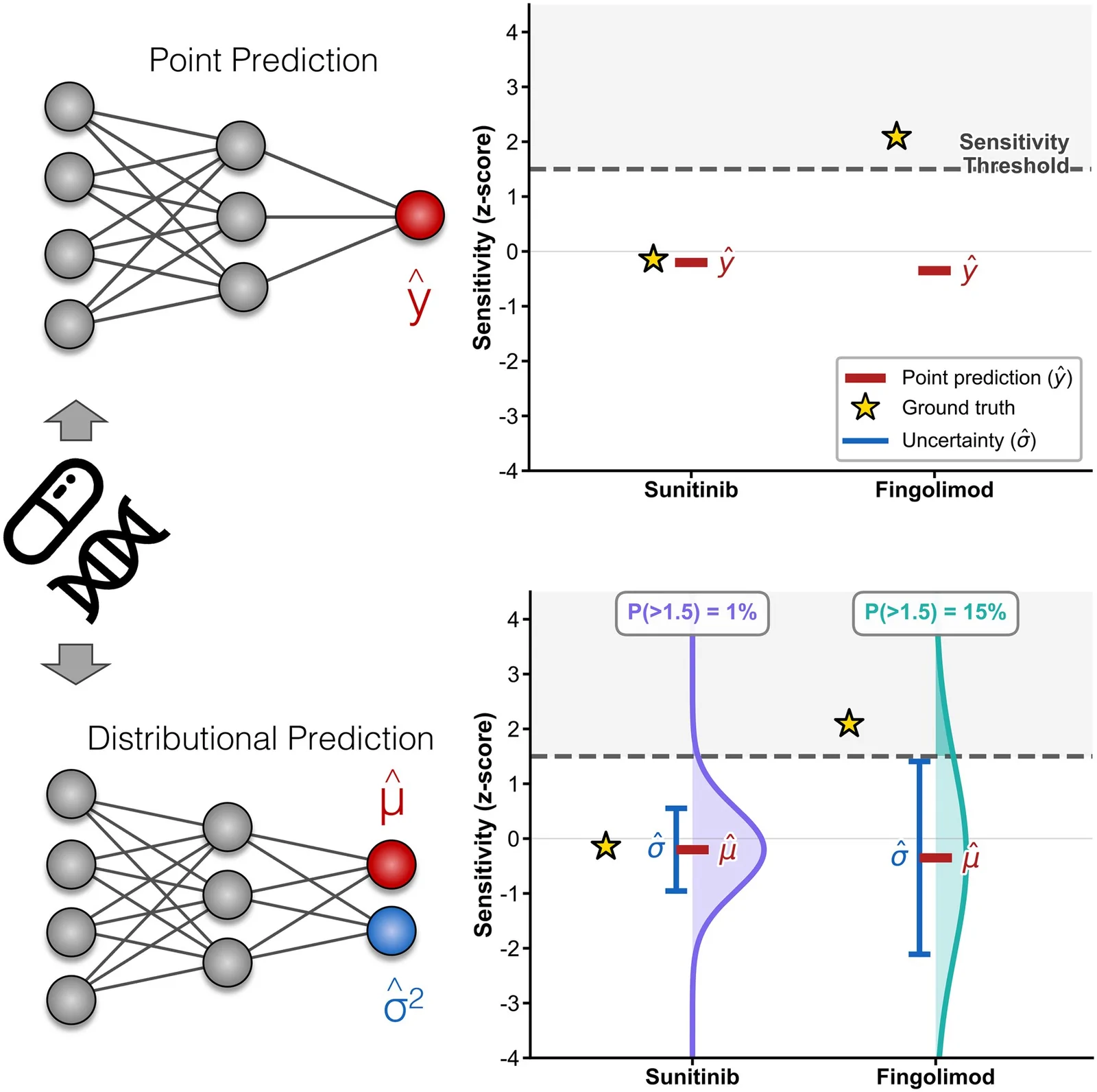
Uncertainty estimates enable more informative drug candidate analyses. Predicted means versus predicted distributions from a Gaussian Neural Network Ensemble for two drugs in the leukemia cell line CESS. Fingolimod has a lower mean (point) prediction but higher uncertainty, placing more probability mass above a drug-wise scaled relative sensitivity threshold at *z* = 1.5, i.e. more sensitive than approximately 93% of cell lines for that drug. Responses (sensitivities, −log IC50) are *z*-scored per drug. Stars: ground truth relative drug sensitivity. Example selected for illustration. For more predictions for this cell line, see Fig. S2, available as supplementary data at *Bioinformatics* online.

Explicitly modeling uncertainty can ensure that ML-based cancer drug sensitivity models become more useful and reliable. Uncertainty can indicate when to trust a prediction or when additional validation is needed. Further, uncertainty estimates allow a model to restrict its predictions to high-confidence drugs: It may be more important to find one highly effective candidate drug which is predicted with a high confidence, instead of accurately representing the drug response of all available compounds. Conversely, uncertainty enables probabilistic comparisons between predictions. A drug with a moderately unfavorable predicted mean response but high uncertainty may place more probability mass in relevant regions than one with a better mean response but a narrow uncertainty band (see Fig. 1 for an example).

Uncertainty quantification is an active area of research. Approaches range from Bayesian methods and frequentist inference to evaluating the agreement of ensembles. It is not *a priori* known which method is superior for a given dataset, as they differ in their assumptions, computational cost, and suitability to the model and data at hand (Abdar *et al*. 2021, Fakour *et al*., 2024). However, comprehensive benchmarks that jointly assess predictive accuracy and the reliability of uncertainty estimates for drug response prediction are lacking. To address this, we benchmark various uncertainty-aware ML approaches along these two axes and discuss strategies for their joint evaluation. Uncertainty estimation also opens up downstream applications that we showcase, such as detecting transcriptomic drivers of uncertainty, i.e. genes whose expression is associated with unpredictability of the drug response rather than sensitivity or resistance. These genes may characterize biological states that lead to variable drug responses and would be missed in standard explainability analyses. We further demonstrate that uncertainty estimates can guide active learning to propose informative measurements.

### 1.1 Related work

Extensive research has been dedicated to identify the best point predictors for drug response (Adam *et al.* 2020), but the community has given little attention to leveraging uncertainty quantification methods in this domain (Lenhof *et al.* 2024a). Fang *et al.* (2018) and Nolte *et al*. (2024) propose quantile and deep regression forests, respectively, for constructing prediction intervals in drug response prediction. Lenhof *et al.* (2024b) focus on applying conformal prediction to achieve reliable drug sensitivity estimation and drug ranking under a novel viability measure. They employ quantile regression forests to estimate the uncertainty in drug-sensitivity regression. Rolli *et al*. (2026) extend this approach with a cluster analysis that scores how far test samples lie from their nearest training samples to assess the limits of the model applicability. Peres da Silva *et al.* (2021) weight task- and domain-level aleatoric and epistemic uncertainty to improve transfer from panels to *in vivo* settings. However, these works do not compare the effectiveness of uncertainty quantification methods for drug response prediction. Further, other methods for uncertainty-aware modeling such as distributional neural networks (Bishop 1994, Koenker and Bassett 1978, Nix and Weigend 1994, Padilla *et al*. 2022), Bayesian inference (MacKay 1992, Gal and Ghahramani 2016), evidential deep learning (Amini *et al*. 2020), or ensembles of neural networks (Lakshminarayanan *et al*. 2017) have not been considered for drug response prediction. In related molecular property prediction tasks, benchmarks have found optimal uncertainty quantification methods to be highly dataset-, split-, and task-specific, underscoring the need for domain-specific systematic benchmarking (Hirschfeld *et al*. 2020, Greenman *et al.* 2025).

## 2 Methods

In this work, we compare methods for quantifying uncertainty in drug response prediction and apply these estimates to downstream analytical tasks. First, we evaluate seven uncertainty-aware drug response models on point prediction accuracy and uncertainty quality. Second, we test the ability of these uncertainty estimates to detect out-of-distribution inputs under dataset shifts and generalization settings. Third, we leverage the generated uncertainty estimates for model interpretation and active learning. The code for all experiments, analyses, and figures is available at https://github.com/PascalIversen/LUDRP. The input data to run the code is available at https://zenodo.org/records/19219090.

### 2.1 Data

We train and evaluate the models on logarithmic IC50 drug response data from the publicly available GDSC dataset. We *z*-scale the log IC50 values over all drug-cell line pairs for each fold using statistics calculated on the train splits. We use mRNA expression data as cell line features, which are hypothesized to have the highest predictive power (Manica *et al*. 2019). Following Manica *et al.* we use expression from 2089 genes selected by network diffusion from the STRING protein-protein interaction network around drug targets. We process GDSC’s microarray mRNA expression data using an arcsinh transformation, followed by gene-wise standardization (*z*-scoring) based on statistics computed from the training set (Manica *et al*. 2019). We use RDKit (https://www.rdkit.org/) to encode the chemical structure of the anti-cancer drugs as 128-bit Morgan fingerprints (Rogers and Hahn 2010). After filtering cell lines and drugs with missing gene expression values or drug fingerprints, respectively, we are left with 924 cell lines, 367 drugs, and 292 165 IC50 values. To test whether our main findings are specific to GDSC and for cross-dataset experiments, we additionally reproduce the benchmark findings on the independent Cancer Therapeutics Response Portal (CTRPv2) (Seashore-Ludlow *et al*. 2015) screen with Broad’s CCLE RNA-seq mRNA expression data (Ghandi *et al.* 2019), which after the same filtering comprises 823 cell lines, 466 drugs, and 180 490 logarithmic IC50 values. We further use BeatAML (Tyner *et al*. 2018) for cross-dataset experiments, a dataset of *ex vivo* drug responses in primary samples from acute myeloid leukemia (AML) patients. After restricting to the 80 drugs it shares with CTRPv2 and to samples with available RNA-seq gene expression, it contains 476 samples and 10 017 IC50 values.

### 2.2 Benchmark

#### 2.2.1 Uncertainty in drug response prediction

Commonly, drug response prediction is considered as a regression task (Adam *et al.* 2020): Given a set of cell lines and drugs indexed by $i=1,\ldots ,N_{c}$ and $j=1,\ldots ,N_{d}$, respectively, we observe drug response values $y_{ij}\in R$ (e.g. log IC50) for a subset of samples (i,j)∈O, where $O\subseteq \{1,\ldots ,N_{c}\}\times \{1,\ldots ,N_{d}\}$ denotes the index set of observed cell line–drug pairs. We define $x_{i}^{\left(c\right)}\in R^{p}$ as the feature vector of cell line *i* and $x_{j}^{\left(d\right)}\in R^{q}$ as the feature vector of drug *j*. The training data consists of tuples $D=\{(x_{i}^{\left(c\right)},x_{j}^{\left(d\right)},y_{ij})|(i,j)\in O\}.$

The goal is to learn a predictive function $f:R^{p}\times R^{q}\to R$ such that for any $i,j:\;{\hat{\text{y}}}_{\text{ij}}=f(x_{i}^{\left(c\right)},x_{j}^{\left(d\right)})$ approximates the true drug response $y_{ij}$. This regression task is usually modeled as point prediction, i.e. the models only estimate the mean of the conditional distribution $y_{ij}|(x_{i}^{\left(c\right)},x_{j}^{\left(d\right)})$ of the drug response value by optimizing the Mean Squared Error (MSE) or a similar loss function. The conditional mean provides a narrow representation of the statistical characteristics of the response, and model certainty can only be assessed globally using empirical risk measures, such as the MSE over a test set. This assumes that the residual variance is approximately homoscedastic over the feature space. In practice, the predictability of a drug-cell line pair’s response can vary significantly depending on the cell line’s molecular state and its interactions with drug characteristics. We consider models that capture higher-order characteristics such as the variance or quantiles of the conditional distribution by explicitly accounting for heteroscedastic prediction errors. These characteristics allow the models to quantify instance-level aleatoric uncertainty in predictions.

Aleatoric uncertainty quantifies the irreducible variability in the drug response $y_{ij}$ for a given cell line-drug pair (i,j) with features $\left(x_{i}^{\left(c\right)},x_{j}^{\left(d\right)}\right)$, arising from inherent randomness in the system. The predicted aleatoric uncertainty is a model-derived estimate of the conditional residual variability of the response given the features. It is not a direct measurement of biological stochasticity but includes sources like measurement noise, dose–response curve-fit error, and assay artifacts. The reliability of aleatoric uncertainty breaks down in out-of-distribution scenarios. These can occur due to underrepresentation of cancer types in the training data, inconsistencies in measurement conditions, or biological shifts such as differences between patient-derived and long-cultured cell lines (Gillet *et al*. 2013). It necessitates estimating epistemic model uncertainty, which quantifies the model’s uncertainty about the predicted parameters of $p(y|x_{i}^{\left(c\right)},x_{j}^{\left(d\right)})$, including, in probabilistic models, the predicted variance. In theory, epistemic uncertainty can be used to detect distribution shift (Kendall and Gal 2017). It can be reduced by collecting more data in underrepresented regions.

#### 2.2.2 Uncertainty-aware models

We evaluate seven modeling approaches to assess how well different paradigms estimate uncertainty in drug response prediction. We select methods that are commonly applied, require minimal uncertainty-specific hyperparameter tuning, and produce a continuous uncertainty score. We include methods that do and do not rely on neural networks, focus on different uncertainty types (epistemic, aleatoric, or both), and span different computational costs, from linear models to neural ensembles. We exclude post-hoc wrappers on existing predictions, such as post-hoc calibrators or conformal prediction, which adjust prediction intervals but do not change the underlying uncertainty ranking (Tibshirani *et al*. 2019). Our selection covers the major families of uncertainty estimation identified in the literature (Abdar *et al*. 2021, Fakour *et al.* 2024): distributional modeling, ensemble methods, Bayesian inference, and evidential learning. While in practice, the distinction between aleatoric and epistemic uncertainty is not perfect (Gruber *et al*. 2025), we categorize methods by their estimation method: distributional modeling for aleatoric, ensembles or Bayesian approaches for epistemic.

We evaluate two neural network-based approaches that estimate aleatoric uncertainty by explicitly modeling the predictive distribution:

We employ a Gaussian Neural Network (GaussNN), assuming a heteroscedastic Gaussian conditional distribution $p\left(y|x_{ij}\right)∼N\left(\mu_{ij},\sigma_{ij}^{2}\right)$, where $x_{ij}=[x_{i}^{\left(c\right)};x_{j}^{\left(d\right)}]\in R^{p+q}$ denotes the concatenated feature vector (Bishop 1994, Nix and Weigend 1994). We predict the distribution using a neural network $f_{\theta }:R^{p+q}\to R\times R^{+}$ with weights θ and two output neurons producing the mean and variance estimates $f_{\theta }\left(x_{ij}\right)=({\hat{\mu }}_{ij},{\hat{\sigma }}_{ij}^{2})$. We minimize the Gaussian negative log-likelihood: $L\propto \sum_{i=1}^{n}\left(\text{log}({\hat{\sigma }}_{ij}^{2})+\frac{{\left(y_{ij}-{\hat{\mu }}_{ij}\right)}^{2}}{{\hat{\sigma }}_{ij}^{2}}\right)$ and interpret the predicted variance as a measure of the aleatoric uncertainty of the model predictions. We train it with Fast Gradient Sign adversarial training (Goodfellow *et al*. 2015, Lakshminarayanan *et al*. 2017) to prevent sharp predictive distributions.

Instead of modeling the output distribution parametrically, Quantile Neural Networks (QuantileNNs) predict specific quantiles, i.e. the model outputs estimates ${\hat{q}}_{\tau }(x_{ij})$ for a chosen quantile level τ∈(0,1) (Koenker and Bassett 1978, Padilla *et al*. 2022). We minimize the quantile loss, which penalizes over- and underestimation of the quantile predictions. We use quantiles τ=0.95, τ=0.50, τ=0.05. We measure the aleatoric uncertainty as the difference between the 0.95 and 0.05 quantile predictions (the width of the 90 % prediction interval).

To quantify epistemic uncertainty, we rely on Bayesian and ensemble-based methods. As a simple baseline, we use a Random Forest (RF). We estimate uncertainty as the variance of the predictions from individual decision trees (Mentch and Hooker 2016). The predictions will vary more if the models are not constrained by training examples similar to the input, and a lack of agreement, therefore, reflects epistemic uncertainty.

We also evaluate Bayesian Ridge Regression (BayesianRidge) (MacKay 1992), a linear model with Gaussian priors over weights and noise. It has a closed-form predictive variance $\text{Var}(y|x_{ij})=x_{ij}^{⊤}\Sigma x_{ij}+\alpha^{-1}$, modeling epistemic uncertainty through the posterior covariance Σ and a homoscedastic component $\alpha^{-1}$.

We further employ an Monte Carlo Dropout Neural Network (MCDropout) (Gal and Ghahramani 2016), which applies dropout at both training and inference time. Multiple forward passes yield samples from an approximate posterior, with the variance quantifying epistemic uncertainty. We use 10 inference passes and train with MSE.

To capture both uncertainty types, we analyze a Gaussian Neural Network Ensemble (GaussNNEns) (Lakshminarayanan *et al*. 2017), where M=10 GaussNNs are trained independently with different initializations. The ensemble mean serves as a point prediction, and the total predictive variance decomposes into an aleatoric component (mean of member variances) and an epistemic component (variance of member means). Mathematical details are provided in the supplement (*Gaussian NN Ensemble variance decomposition*). For this work, we also reduce inter-model correlation by resampling training and early-stopping validation data for each member.

Evidential Deep Learning (EvidentialDL) (Amini *et al*. 2020), which also uses the Gaussian likelihood, places a Normal-Inverse-Gamma prior over the likelihood parameters. The network outputs four parameters $\left(\gamma_{ij},v_{ij},\alpha_{ij},\beta_{ij}\right)$ from which aleatoric uncertainty ${\hat{\sigma }}_{ij,\text{al}}^{2}=\frac{\beta_{ij}}{\alpha_{ij}-1}$ and epistemic uncertainty ${\hat{\sigma }}_{ij,\text{ep}}^{2}=\frac{\beta_{ij}}{v_{ij}(\alpha_{ij}-1)}$ are derived, with ${\hat{\mu }}_{ij}=\gamma_{ij}$ as point prediction. Training maximizes the likelihood with a regularizer that penalizes overconfidence on training examples.

#### 2.2.3 Model training and evaluation

We evaluate using a 5-fold leave-cell-line-out cross-validation to prevent cell line memorization from biasing the performance estimates. This setting reflects the primary deployment scenario of these models: predicting the response of an unprofiled cell line or patient-derived sample to a known panel of drugs. Drug response models currently still extrapolate poorly to unseen drugs (leave-drug-out setting) (Bernett *et al.* 2026). We therefore restrict our uncertainty benchmark to cell-line generalization. For hyperparameter tuning, we split an additional leave-cell-line-out validation set from the train set of each fold and perform hold-out hyperparameter optimization. Details regarding training and hyperparameter search are in the supplement (*Training details*). We assess performance of the mean estimate using the MSE and Pearson correlations between predicted and observed z-scaled logarithmic IC50 values of each cross-validation fold test set. Computing correlation metrics globally across all drugs can inflate performance due to differences in average drug potencies (Bernett *et al.* 2026). We also compute drug-wise aggregated performance metrics: We first join the test sets of all cross-validation folds, compute the metrics stratified by drug, and then report the median and standard deviation over drugs.

To evaluate uncertainties, we assess calibration and sharpness. Calibration measures how well the predicted uncertainty corresponds to the empirical test error frequency, i.e. whether prediction intervals contain the true values at the expected rate. We quantify this using the miscalibration area (MA): We integrate the absolute difference between observed and expected coverage across a grid of nominal prediction-interval levels spanning 0–1. The BayesianRidge, EvidentialDL, GaussNN and GaussNNEns explicitly parameterize a Gaussian predictive distribution. We approximate the outputs of other models as Gaussian, using the mean and standard deviation of predictions across ensemble members (RF, MCDropout), or by converting the interquantile range of the QuantileNN to a standard deviation. This assumption enters only into the calibration and sharpness comparison, where prediction intervals are needed to place all models on a common scale; the area under the uncertainty–reduction curve (AUURC), MSE@*k* % and out-of-distribution results are computed from the predicted uncertainty scores directly and make no distributional assumption; see Fig. S1, available at supplementary material  *Bioinformatics Advances* online for a non-parametric sensitivity check on the RF. Coverage refers to the proportion of true drug response values that fall within a given prediction interval. A well-calibrated model achieves coverage close to the nominal level (e.g. a 90% prediction interval should empirically contain approximately 90% of true values). We measure sharpness by the average width of the 90 % prediction intervals. A well-calibrated model (low MA) with sharper intervals is preferred (Chung *et al*. 2021).

We report ranking-based measures alongside calibration; the reasoning is given in the supplement (*Calibration and post-hoc recalibration*).

We additionally report two measures of uncertainty quality that are independent of the overall point accuracy. First, we compute the Pearson correlation $r(|\epsilon |,\hat{\sigma })$ between the absolute error |ϵ| and the predicted uncertainty $\hat{\sigma }$. Higher correlations indicate better uncertainty quality. Second, we obtain a normalized ranking skill $1-(\text{AUURC}-{\text{AUURC}}_{\text{oracle}})/({\text{AUURC}}_{\text{rand}}-{\text{AUURC}}_{\text{oracle}})$. Here, ${\text{AUURC}}_{\text{oracle}}$ and ${\text{AUURC}}_{\text{rand}}$ are the AUURC obtained when test instances are removed in order of their absolute error (i.e. perfect removal strategy) and at random, respectively. Higher values indicate better skill of predicted uncertainty at distinguishing erroneous predictions.

For out-of-distribution detection, we add to each test feature a signed offset, with the sign drawn uniformly from {−1,+1} per feature and the magnitude drawn from a Gaussian with mean μ and standard deviation $10^{-4}$, for μ∈{0.01,0.1,0.2,…,1.0}, as synthetic perturbations to the test instance features. We additionally evaluate uncertainty under real distribution shifts between screening datasets. We train each model on one screen (CTRPv2, because it relies on RNA-seq also used in the other considered study) and predict on GDSC or BeatAML. We restrict to drugs shared between the two screens and to cell lines not seen during training. We consider three transfers with different feature shifts: (i) To GDSC response data but using Broad’s CCLE RNA-seq data as features. This removes the microarray-versus-RNA-seq platform difference and leaves a biological/population shift. (ii) To the BeatAML patient extracted acute-myeloid-leukemia dataset. BeatAML uses an independent RNA-seq screen from a different laboratory, but the same sequencing platform, introducing a cross-laboratory and biological shift. (iii) To GDSC using its native RNA microarray gene expression features. This constitutes a strong technical platform shift for the input features, in addition to the biological/population and laboratory shift. For each synthetic perturbation level and for each cross-dataset prediction, we pool the in-distribution test instances of all folds with the same count of matched out-of-distribution instances and use the predicted uncertainties as scores to classify each instance as out-of-distribution (high predicted uncertainty) or in-distribution (low uncertainty). We evaluate this classification via area under the receiver operating characteristic curve (AUROC). Higher values indicate stronger separation of shifted samples based on uncertainty.

### 2.3 Applications of uncertainty

#### 2.3.1 Explaining uncertainty

Current explainability approaches in drug response modeling attribute the predicted mean response to input features (Kuenzi *et al*. 2020, Tang and Gottlieb 2021, Shi *et al.* 2025). By leveraging uncertainty estimates, we can also detect drivers of predictive uncertainty. In the GaussNN, both the mean and the variance are direct outputs of the model, and we can estimate Shapley values for both outputs (Iversen *et al*. 2025). The attribution of uncertainty highlights features that act as uncertainty drivers. These features affect the models’ uncertainty estimation without necessarily influencing the predicted mean response.

#### 2.3.2 Uncertainty-guided sample-specific fine-tuning

To evaluate whether uncertainties can be of benefit in an active learning scenario, we simulate a preclinical screening scenario in which a new cell line or patient-derived sample is added to a study. Under a limited measurement budget, the goal is to measure the drugs that maximize the information gain for the IC50 profile of a cell line or patient-derived model. This especially reflects constraints in organoid or PDX studies, where comprehensive drug screening is expensive (Nikeghbal *et al*. 2025). We pretrain a GaussNN model on a split with held-out cell lines in the test set. For each held-out cell line, we simulate measurements by selecting a set of 60 drug response observations for this cell line. We choose the drugs with the highest predicted uncertainty for that cell line as estimated by the pretrained model. As a baseline, we sample the drugs randomly from the available set. These additional drug response measurements are then combined with the original training data, and the model is fine-tuned on one of these datasets with early stopping and up to 100 epochs with the Adam optimizer defaults. We evaluate the model performance on the full set of drug responses, including the measured ones, for the held-out cell line using the MSE to measure the information gain compared to the baseline. Further, we evaluate on the subset of drugs not included in the fine-tuning process of either method to assess the relative generalization performance increase of the baseline versus the uncertainty-guided selection.

## 3 Results

### 3.1 Benchmark

We evaluate seven models estimating drug response and aleatoric (QuantileNN, GaussNN), epistemic (RF, BayesianRidge, MCDropout), or both uncertainty types (GaussNNEns, EvidentialDL). Experiments use 5-fold leave-cell-line-out cross-validation on GDSC (924 cell lines, 367 drugs, 292 165 pairs).

#### 3.1.1 Predictive and uncertainty performance is highest for GaussNN variants

We evaluate all models in two dimensions: predictive accuracy on unseen cell lines and the quality of their uncertainty estimates. For predictive accuracy, Table 1 reports the MSE and Pearson correlation between point predictions and ground truth. Metrics are reported either drug-wise (after merging folds, computed separately for each drug and then aggregated with the median) or globally (computed over all drug-cell-line pairs of each fold and then aggregated with the median). The reported standard deviations reflect variability across drugs or folds. The GaussNN variants and RF significantly outperform all other methods for global Pearson and MSE (paired two-sided t-tests, Benjamini-Hochberg corrected, for details see Fig. S3, available at supplementary material  *Bioinformatics Advances* online). BayesianRidge consistently underperforms across all metrics, likely because of its linear model assumption. Overall, the Pearson correlations drop substantially when evaluated at the per-drug level. The large variation in overall drug potencies inflates correlations in the global setting (Bernett *et al.* 2026).

**Table 1:** MSE and Pearson correlation between model predictions and experimental logarithmic IC50 values on GDSC data.

	Random Forest.	Bayesian Ridge.	Quantile NN.	MC Dropout NN.	Gaussian NN.	Gaussian NN Ens.	Evidential DL.
***Drug-wise***							
MSE	**0.187** ± 0.128	0.446 ± 0.596	0.221 ± 0.145	0.222 ± 0.141	0.202 ± 0.138	0.197 ± 0.141	0.222 ± 0.150
Pearson	0.425 ± 0.121	0.323 ± 0.127	0.331 ± 0.123	0.331 ± 0.122	0.382 ± 0.128	**0.435** ± 0.127	0.325 ± 0.121

***Global***							
MSE	**0.220** ± 0.005	0.613 ± 0.005	0.250 ± 0.011	0.255 ± 0.003	0.237 ± 0.006	0.235 ± 0.004	0.255 ± 0.006
Pearson	**0.883** ± 0.002	0.624 ± 0.004	0.867 ± 0.006	0.864 ± 0.002	0.876 ± 0.003	0.881 ± 0.002	0.864 ± 0.003

For the drug-wise metrics, values are the median over drugs; the standard deviation represents the variability across drugs. The global metrics are calculated for the folds, and the standard deviation is the variation over folds. Best values are shown in bold; second-best are underlined. For results on CTRPv2 see Table S1, available as supplementary data at *Bioinformatics* online.

**Figure 2:**
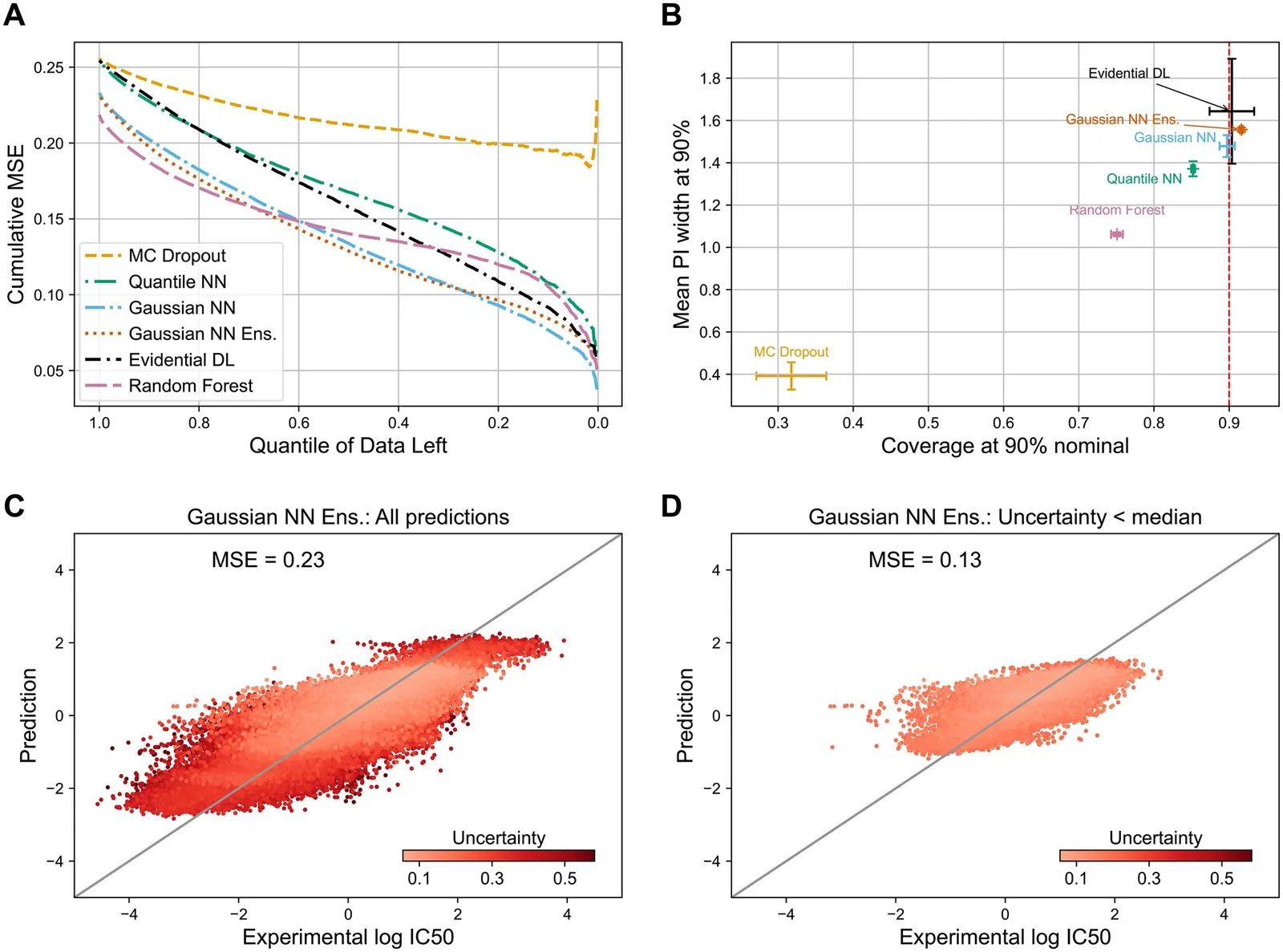
Uncertainty-based filtering, calibration and sharpness across models. (A) Uncertainty-based filtering curves. The *x*-axis shows the fraction of test samples retained after thresholding by predicted uncertainty (most uncertain removed first, left to right). The *y*-axis reports the MSE computed on the reduced test subset. A steeper decline indicates a more effective uncertainty estimation. (BayesianRidge left out for visibility, see Fig. S4, available as supplementary data at *Bioinformatics* online) (B) Calibration–sharpness tradeoff across models. Each point is the mean empirical coverage and average prediction interval (PI) width at 90 % nominal coverage. Error bars indicate variability across folds. (C) Observed versus predicted log IC50 values for the GaussNNEns on all test samples, colored by predicted uncertainty. (D) Same as (C), but restricted to the 50 % most confident predictions.

We evaluate the informativeness of the uncertainty estimates by how well the model trades off coverage of the input space suitable for predictions with the model against accuracy. Figure 2A shows uncertainty reduction curves, also referred to as confidence curves (Rasmussen *et al*. 2023), for all models. Each point corresponds to the MSE obtained after iteratively removing the cell-line drug pairs in the test set with the highest uncertainty as predicted by the model and re-evaluating the MSE on the remaining samples. For informative uncertainty estimates, the curve falls monotonically (low AUURC) and achieves low MSE@*k* % at small values of *k*. By contrast, an uninformative estimate yields a near-flat trajectory, as also expected from a random order of test points. We find that the uncertainty of the BayesianRidge is uninformative, as the curve is nearly flat (see Fig. S4, available at supplementary material  *Bioinformatics Advances* online). All other tested models exhibit a decline, indicating that higher predicted uncertainty corresponds to higher error. The GaussNN variants achieve the largest reduction. Restricting predictions to the 10 % most confident samples reduces the global MSE by 64 % (from 0.23 to 0.08) for the GaussNNEns. This reduction is not simply driven by excluding predictions far from the population mean (Fig. S5, available at supplementary material  *Bioinformatics Advances* online). The predictions of the GaussNNEns are depicted in Fig. 2C and D, with and without restricting by uncertainty to below the median.

Table 2 summarizes the uncertainty ranking metrics AUURC and MSE@*k* % across models. The GaussNN variants achieve similar results and significantly outperform all other model types, attaining the lowest AUURC, MSE@10 %, and MSE@50 % (see Fig. S6, available at supplementary material  *Bioinformatics Advances* online for statistical test results). While the RF is a comparatively strong point predictor, on GDSC it trails the GaussNN variants on AUURC and MSE@*k* %. Overall, the GaussNN variants achieve the lowest MSE@10 %. The RF, EvidentialDL, and QuantileNN clearly outperform the MCDropout and BayesianRidge. For example, the MCDropout and the QuantileNN start from a similar baseline MSE (QuantileNN 0.250, MCDropout 0.255; Fig. 2A). Owing to better uncertainty estimation, on the 10 % most confident predictions the QuantileNN outperforms the MCDropout by 44 % (0.108 versus 0.192). The ranking skill confirms this across models: the RF and GaussNN variants are similarly accurate point predictors (Table 1), yet the RF reaches only 0.42 ranking skill, whereas the GaussNN variants rank errors best (0.51–0.52). The per-instance correlations between absolute error and uncertainty, $r(|\epsilon |,\hat{\sigma })$, are consistent with this, delivering highest values for GaussNN variants. Note that these are modest by construction: even with perfect uncertainty estimation, the model error is a single realization drawn from the predictive distribution. Figure 2B compares the empirical coverage as a measure of uncertainty calibration and mean width of 90 % prediction intervals as a measure of sharpness of uncertainty estimates across models. Coverage close to a chosen target level (we depict the target level 90 %) with narrow prediction intervals indicates good calibration and sharpness. The GaussNN and its ensemble, as well as EvidentialDL achieve coverage near the nominal target. The calibration of these models is stable over different levels, as they achieve a low MA (see Fig. S7, available at supplementary material  *Bioinformatics Advances* online). MCDropout and RF yield the sharpest intervals; however, only at the cost of overconfidence: Their prediction intervals contain a smaller proportion of true values than intended.

**Table 2:** Uncertainty evaluation metrics per model on GDSC data (average ± standard deviation over the cross-validation folds).

	Random Forest.	Bayesian Ridge.	Quantile NN.	MC Dropout NN.	Gaussian NN.	Gaussian NN Ens.	Evidential DL.
AUURC	0.143 ± 0.002	0.599 ± 0.003	0.168 ± 0.013	0.213 ± 0.008	0.136 ± 0.004	**0.135** ± 0.003	0.156 ± 0.006
MSE@10 %	0.105 ± 0.002	0.597 ± 0.025	0.108 ± 0.013	0.192 ± 0.011	**0.077** ± 0.006	0.084 ± 0.005	0.089 ± 0.006
MSE@50 %	0.140 ± 0.002	0.600 ± 0.008	0.169 ± 0.013	0.209 ± 0.008	0.133 ± 0.004	**0.129** ± 0.002	0.155 ± 0.008
Ranking skill	0.42 ± 0.02	0.03 ± 0.01	0.42 ± 0.03	0.21 ± 0.03	0.51 ± 0.02	**0.52** ± 0.01	0.48 ± 0.02
$r(|\epsilon |,\hat{\sigma })$	0.30 ± 0.01	0.02 ± 0.01	0.26 ± 0.02	0.14 ± 0.02	0.33 ± 0.01	**0.35** ± 0.01	0.29 ± 0.02
MA	0.10 ± 0.01	0.28 ± 0.00	0.03 ± 0.00	0.34 ± 0.02	**0.01** ± 0.01	0.02 ± 0.00	0.03 ± 0.03

AUURC, area under the uncertainty–reduction curve (lower = better). MSE@*k* %: MSE on the *k* % most confident predictions (lower = better). MA, miscalibration area (lower = better calibrated). Ranking skill, normalized risk–reduction skill, independent of overall accuracy (1 = oracle error ordering, 0 = random; higher = better). $r(|\epsilon |,\hat{\sigma })$, Pearson correlation between absolute error and predicted uncertainty (higher = better). Best values are shown in bold, second-best underlined. For results on CTRPv2 data, see Table S2, available as supplementary data at *Bioinformatics* online.

These results also largely hold on CTRPv2 (Tables S1 and S2, available at supplementary material  *Bioinformatics Advances* online): the GaussNN Ensemble again ranks errors best (highest ranking skill and $r(|\epsilon |,\hat{\sigma })$). However, the RF is the strongest point predictor on CTRPv2 and, in contrast to GDSC, its tree-variance uncertainty is well calibrated for in-distribution prediction (MA ≈ 0.01).

#### 3.1.2 Ensemble agreement is key for out-of-distribution detection

**Figure 3:**
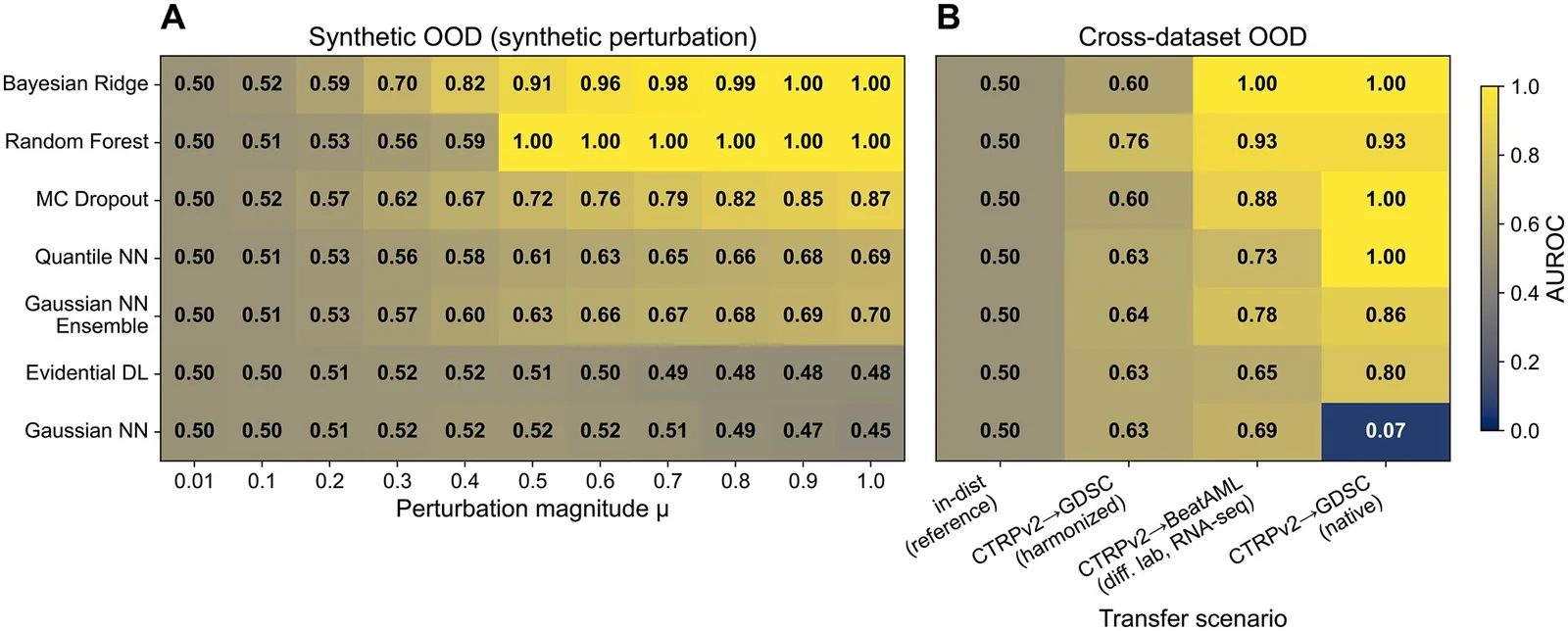
Ensemble-based uncertainty estimates detect distribution shift. Out-of-distribution detection: AUROC for distinguishing in- from out-of-distribution samples based on predicted uncertainty. (A) Synthetic shift: perturbations of increasing magnitude added to the GDSC test features. (B) Real cross-dataset shifts of increasing severity for a CTRPv2-trained model on CCLE RNA-seq gene expression features that predicts cross-dataset with leave cell-line out split. First column: in-distribution reference. Second column: prediction on GDSC data using Broad’s CCLE RNA-seq expression features for harmonizing both datasets (biological/population difference only). Third column: on BeatAML, a different-laboratory RNA-seq cohort (same sequencing platform but different lab, and leukemia patient-extracted versus cancer cell line samples). Fourth column: on GDSC samples with their native RNA microarray gene expression features (strong technical platform shift with biological/population difference). Model errors also rise with the increasing shift (Table S3, available at supplementary material  *Bioinformatics Advances* online). Epistemic uncertainties (for example, RF, BayesianRidge, MCDropout) are more reliable OOD detectors than aleatoric estimators. For standard deviations of the synthetic perturbations, see Supplementary Figure S9, available at supplementary material  *Bioinformatics Advances* online.

We use the AUROC to quantify how well uncertainty scores distinguish out-of-distribution (OOD) from in-distribution samples (higher values indicate better separation). As seen in Figs 3A and S8, available at supplementary material  *Bioinformatics Advances* online, the epistemic component of the GaussNNEns, and BayesianRidge are the most responsive to small synthetic perturbations and show an increase for larger shifts. MCDropout shows a similar but smaller increase. In contrast, RF tree variance remains mostly constant until a sudden rise at shift levels beyond 0.4, potentially due to the fixed thresholding in the decision trees. Aleatoric uncertainty estimates (e.g. QuantileNN, GaussNN) are considerably less affected by the shifts. To analyze more realistic OOD-scenarios, we use a CTRPv2-trained model with RNA-seq-based gene expression features on other screens. The detection AUROC rises with the severity of the real shift (Fig. 3B). The strongest shift is the RNA-seq-microarray platform difference, which is detected by all models except for the GaussNN. BeatAML also natively uses RNA-seq, so the shift is less severe and represents laboratory differences and a biological shift to patient-extracted leukemia samples, and is flagged by all models. Harmonizing CTRPv2 and GDSC drug response data onto shared RNA-seq gene-expression features leaves only biological/population differences, which is weakly flagged (AUROC ≈0.6). The prediction errors increase along with the uncertainty (Table S3, available at supplementary material  *Bioinformatics Advances* online). Apart from the single GaussNN, the predicted uncertainties respond to cross-dataset shifts and scale with the shift severity.

Among the tested models, the GaussNNEns best combines high predictive accuracy with calibrated aleatoric uncertainty and an epistemic component that is responsive to distributional change. However, training and inference times scale linearly with the ensemble size, making the GaussNNEns an order of magnitude slower to train than the single GaussNN (for computation time comparisons, see Table S4, available at supplementary material  *Bioinformatics Advances* online).

### 3.2 Applications

Uncertainty-aware models enable probabilistic statements about individual predictions that go beyond point estimates. For example, rather than comparing drugs by their predicted mean response alone, one can evaluate candidates by their probability of exceeding a relevant sensitivity threshold (Figs 1 and S2). We examine further uses of uncertainty estimates below.

#### 3.2.1 Uncertainty varies systematically across tissues and drug target pathways

**Figure 4:**
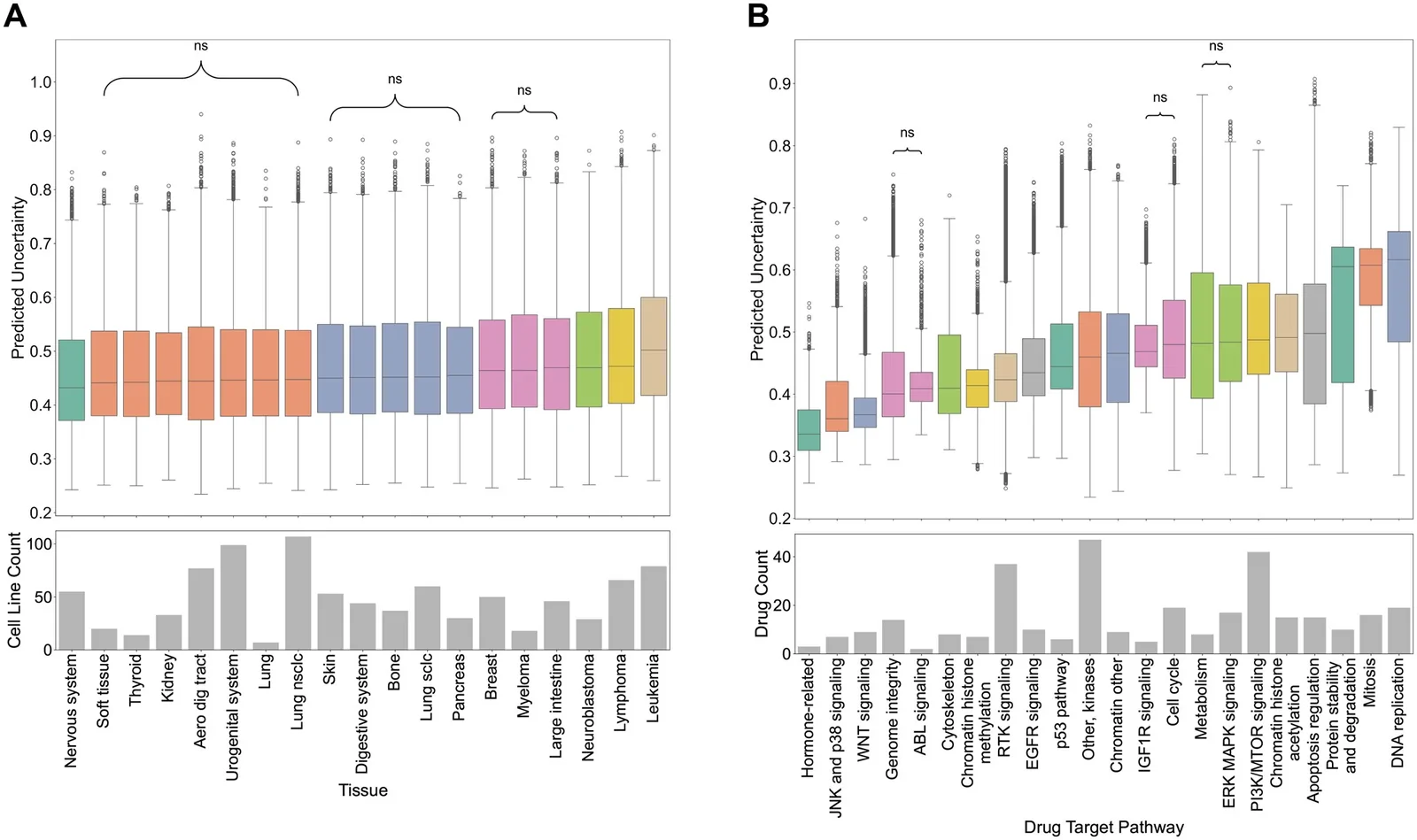
Predictive uncertainty varies across tissues and drug target pathways. Comparison of average predictive uncertainty across (A) tissues and (B) drug target pathways of the GaussNNEns on GDSC data. Statistical differences between groups were assessed using post hoc pairwise Dunn’s test with Holm correction. Groups with non-significant pairwise differences are marked with ‘ns’ and the same boxplot color. Lower panels show sample counts per group. Predictions for hematopoietic malignancies and drugs targeting DNA replication exhibit the highest uncertainty mean. For a version of this figure with decomposed uncertainties (epistemic, aleatoric) see Figure S10, available as supplementary data at *Bioinformatics* online.

We investigate the potential for uncertainty estimates to provide biological insights and inform experimental design. Our analysis focuses on the GaussNN variants, which achieved the lowest overall error and most informative uncertainty estimates, trained on GDSC data. First, we examine whether predictive uncertainty varies systematically across biological and pharmacological subgroups. Figure 4 shows the distribution of the predictive uncertainty for the GaussNNEns stratified by tissue type (A) and drug target pathway (B). We observe significant differences between some tissue types (Kruskal–Wallis to test for overall differences, followed by Dunn’s test with Holm correction for pairwise comparisons). For example, the predictions in cell lines derived from hematopoietic and lymphoid tissue tumors, lymphoma and leukemia, exhibit the highest uncertainties on average, suggesting that these tissue types are more challenging to model. Notably, they are not underrepresented in the training data (Fig. 4 bottom). Instead, their high uncertainty may reflect aleatoric noise or the high intra-class heterogeneity (O’Connor and Tobinai 2014). We observe a similar pattern for the drug-target pathway stratification. The highest median uncertainty is predicted for drugs that target the well-represented class of DNA replication (e.g. Gemcitabine, Bleomycin). In contrast, hormone-related drugs (Bicalutamide, Tamoxifen, AZD3514) are underrepresented but associated with lower predictive uncertainty, possibly due to clearer molecular determinants of response (e.g. estrogen receptor, ESR1, or androgen receptor, AR, expression levels). We analyze whether these patterns are artifacts of the underlying dose-response assay. The uncertainty does correlate with some assay quality metrics such as curve fit $R^{2}$; however, this effect largely disappears once the response is accounted for, as the assay metrics are proxies for the response (Fig. S11, available at supplementary material  *Bioinformatics Advances* online).

**Figure 5:**
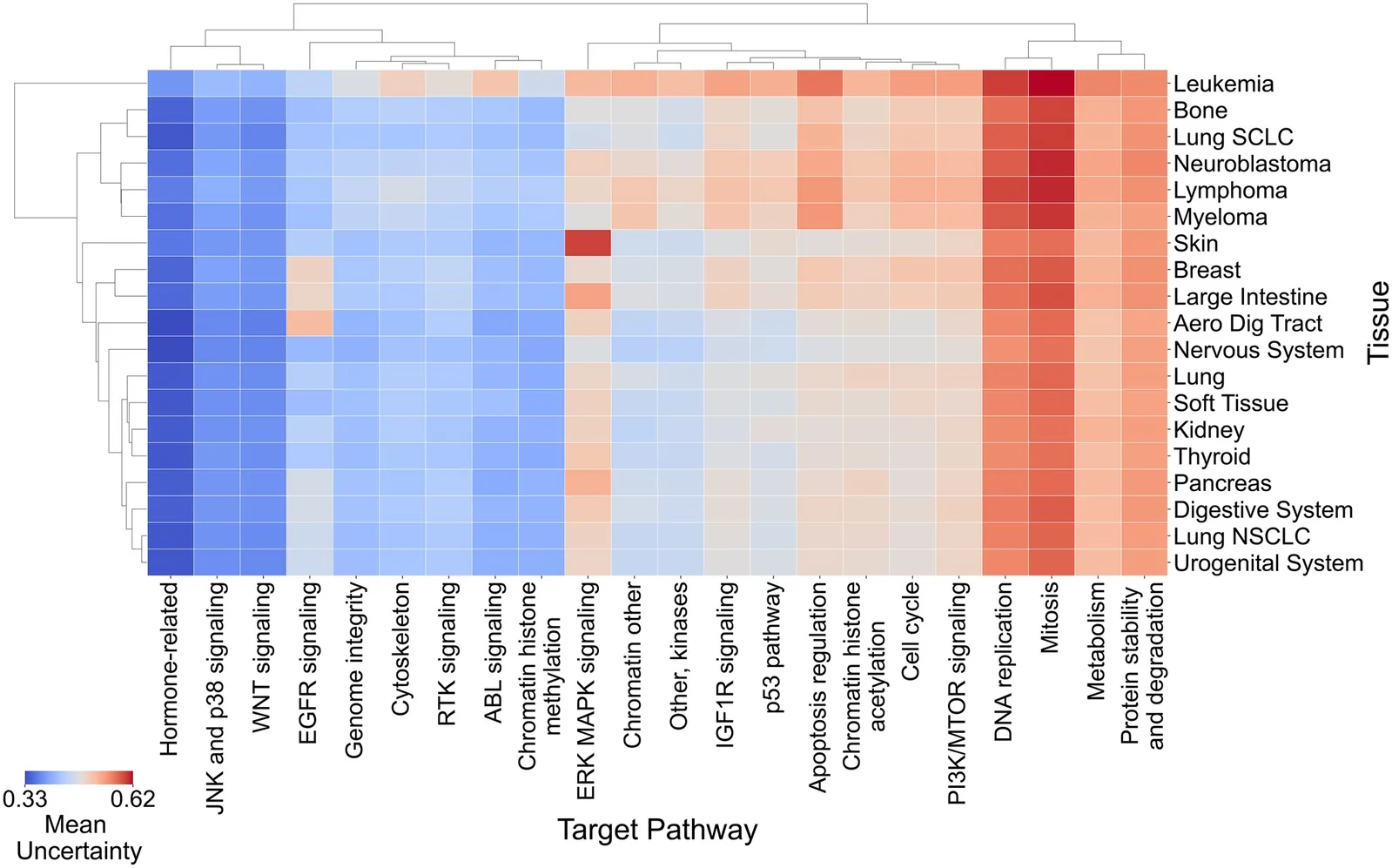
Uncertainty is elevated for specific tissue–pathway combinations. Mean predictive uncertainty across tissues and target pathways of the GaussNNEns on GDSC data, hierarchically clustered in both dimensions. Certain tissue–pathway pairs, such as ERK–MAPK signaling drugs on skin cell lines, show elevated uncertainty relative to other pairs involving these entities.

Further, we form a tissue by pathway matrix of mean predictive uncertainty and apply hierarchical clustering (Fig. 5). This analysis shows similarities between tissues and pathways, and notable outliers. Drugs affecting the ERK-MAPK signaling pathway (e.g. Selumetinib, Refametinib) are associated with high uncertainty for skin cancers. A high prevalence of ERK–MAPK pathway alterations in melanomas, particularly BRAF mutations, is known to give rise to diverse molecular subtypes with heterogeneous responses to drugs targeting ERK-MAPK (Corrales *et al*. 2022).

#### 3.2.2 Uncertainty attribution reveals transcriptomic drivers of uncertainty

**Figure 6:**
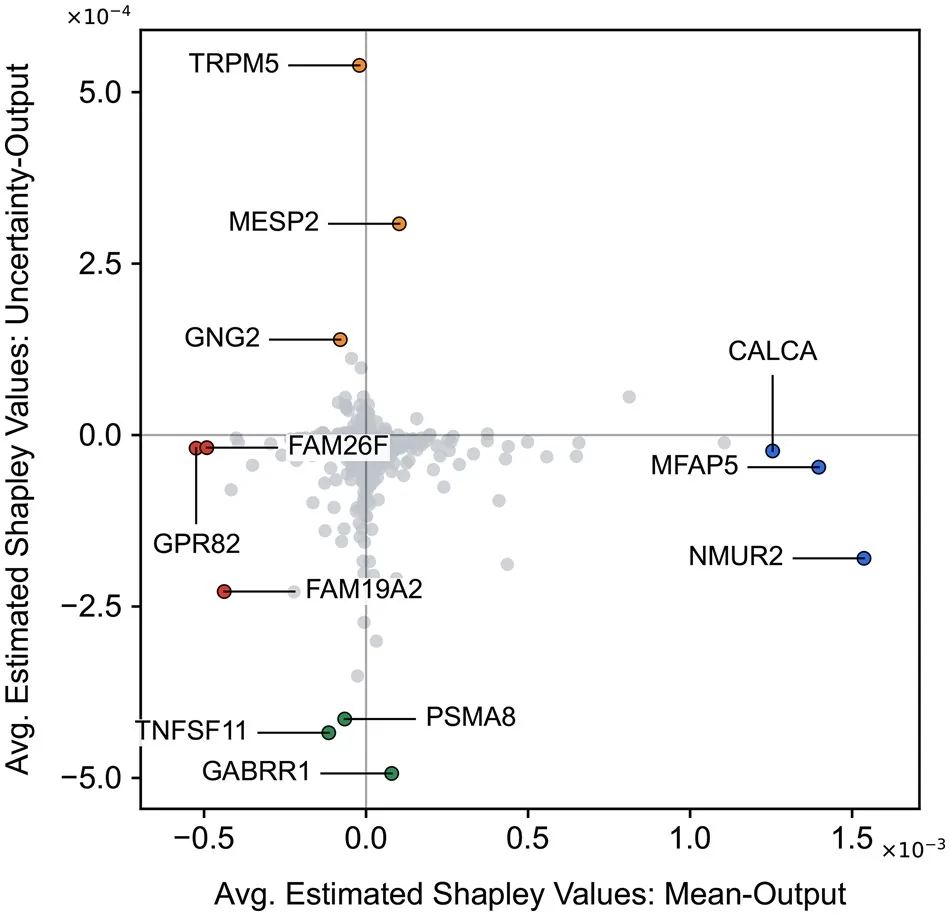
Distinct genes drive the predicted mean response and the predicted uncertainty. Estimated Shapley values of gene-expression features for predicted mean drug response and predictive uncertainty for the GaussNN on GDSC data averaged across the test set. The strongest average contributions, driving weak response, strong response, uncertainty, and certainty are marked in blue, red, orange, and green, respectively. Notably, some genes like TRPM5 and GABRR1 barely influence the mean predicted drug response but strongly affect the uncertainty estimate.

In Fig. 6, we show the averaged mean and variance attributions of the transcriptomic input over all test instances of one cross-validation fold for the GaussNN on GDSC data. Here, genes with positive attribution on the mean output, such as the extracellular regulators of calcium-dependent signaling CALCA, MFAP5, and NMUR2, drive predictions towards resistance. In contrast, sensitivity is associated with FAM26F, GPR82, and FAM19A2, which are also part of the extracellular communication network. Genes with positive attributions for the uncertainty output, for example, signaling genes TRPM5, MESP2, and GNG2, act as drivers of unpredictability. Conversely, the genes PSMA8, TNFSF11, and GABRR1 that regulate tissue-specific functions have strong negative attributions and can be interpreted as stabilizing features. Notably, TRPM5, a transient receptor potential channel linked to chemotherapy resistance and poor prognosis in some cancers, only marginally affects the mean drug response prediction but strongly influences uncertainty estimation (Hu *et al*. 2023, Maeda *et al*. 2017). This modulation of uncertainty would not be captured in analyses restricted to mean effects, and provides a basis for mechanistic hypotheses about unpredictability of drug response (see also Fig. S12, available at supplementary material  *Bioinformatics Advances* online for a comparison of the top contributors to mean and uncertainty outputs). This attribution approach serves as a general framework that can be applied in a highly targeted manner to any tissue–pathway combination of interest. As an example, we contrast leukemia and nervous system cell lines treated with ERK-MAPK-targeting drugs (Fig. S13, available at supplementary material  *Bioinformatics Advances* online). This analysis yields distinct gene-level drivers of both mean response and uncertainty. It allows for the generation of context-specific mechanistic hypotheses.

#### 3.2.3 Uncertainty can inform active selection of informative measurements

We assess whether predictive uncertainty can guide the sample-specific acquisition of informative measurements to fine-tune drug response models. This setting reflects scenarios in which drug response measurements are costly, as in organoid or PDX experiments, and only a limited number of compounds can be screened for a new sample. On the full drug set, including measured drugs, uncertainty-guided fine-tuning significantly reduces MSE compared to random selection (mean MSE 0.216 versus 0.224, $p<10^{-16}$, rank-biserial r=0.60, two-sided Wilcoxon signed-rank test, n=920, see Fig. S14, available at supplementary material  *Bioinformatics Advances* online). This result is consistent with the greedy strategy targeting the most uncertain samples, confirming that the uncertainty estimates meaningfully identify where the model has the most to gain. A small improvement persists even when evaluation is restricted to the intersection of drugs not used for fine-tuning for both approaches (mean MSE 0.183 versus 0.184, $p=2\times 10^{-4}$, rank-biserial r=0.14, n=920). Research into balancing this exploitative strategy with diversity-oriented sampling could lead to greater improvements in this setting.

## 4 Discussion

We benchmark several models that predict drug response along with their predictive uncertainty. We find that distributional modeling methods (QuantileNN, GaussNN) are effective for ranking in-distribution errors of drug-cell line pairs. However, they are unresponsive to distributional shift, and, therefore, their uncertainty estimates are unreliable in settings involving possible domain shifts. Ensemble-based approaches (MCDropout, RF) complement this: While their uncertainty estimates are considerably less precise at ranking errors, they can signal out-of-distribution inputs, indicating the boundaries of the model’s applicability. Combining distributional modeling with ensembling (e.g. GaussNNEns) can provide both advantages, albeit with a significantly higher computational cost. It is the only calibrated model with a functional epistemic component. EvidentialDL promises a single-model alternative that captures both uncertainty types, but it achieved only moderate uncertainty-based error ranking performance and failed to detect distribution shifts, possibly due to its hyperparameter sensitivity. Taken together, our results suggest matching the uncertainty method to the application: distributional models when predictions stay within a single screening panel with consistent measurements, and ensembles when models must generalize across datasets, laboratories, or assay platforms, where batch effects and differences in experimental protocols can introduce distributional shifts.

Consistent with our recent benchmark (Bernett *et al.* 2026), RF is the strongest point predictor. Its tree-variance uncertainty, however, trails the GaussNN variants on uncertainty evaluation metrics on GDSC and detects out-of-distribution inputs only for large perturbations. This confirms that response prediction accuracy and uncertainty quality are largely orthogonal. The gap is dataset-dependent: the RF’s tree-variance uncertainty is overconfident on GDSC but well calibrated on CTRPv2. A large body of work has explored advanced modeling approaches for drug response prediction, but the evaluations focus on point prediction accuracy. We recommend assessing performance in two dimensions: accuracy and uncertainty quality (e.g. using AUURC and MSE@*k* %). For our experiments, all neural network models used simple expression- and fingerprint-based feed-forward architectures (GaussNN, GaussNNEns, QuantileNN, MCDropout). Whether more complex architectures, additional modalities, or prior knowledge can improve uncertainty quality and expose apparent aleatoric uncertainty as epistemic remains to be studied.

Modeling uncertainty is not only a measure of prediction reliability but facilitates new downstream applications. It can be used to probabilistically analyze predictions and aggregating uncertainties by tissue and pathway reveals systematic differences between tissues and drug classes. This can guide the allocation of future screening efforts: cancer types with consistently high uncertainty are recommended for expanded profiling. The ability to attribute uncertainty separately from mean predictions opens a new dimension for generating mechanistic hypotheses. Traditional explainability approaches in the drug response field identify genes associated with sensitivity or resistance, but attributing uncertainty can identify genes that drive (un)predictability. Their expression may characterize biological states that lead to variable or context-dependent drug responses. This could inform mechanistic follow-up studies to study the sources of response variability. The identified genes are distinct from standard sensitivity markers and provide orthogonal information that would be missed in analyses restricted to the mean response.

Finally, uncertainties can be used to prioritize measurements for sample-specific fine-tuning of drug response models, and lead to small but significant gains over a random selection strategy. We expect that strategies that balance exploitation based on uncertainty with exploration of the feature space could yield larger improvements.

Our analysis is subject to limitations. First, we benchmark methods on the widely used GDSC dataset. While we confirm the results for the CTRPv2 dataset, both rely on cell lines which are long-cultured *in vitro* cancer models that differ from primary, patient-extracted cell lines. In addition, we summarize drug sensitivity with the IC50, which yields no direct information about the therapeutic index. Translating uncertainty-aware drug response models into clinical decision support requires several steps beyond this preclinical benchmark. Second, uncertainty-based deferral may preferentially exclude highly sensitive cell line–drug pairs, biasing the retained subset toward easier but less actionable cases. This may also mean that aggregate performance metrics may overstate the ability to identify the most responsive pairs. Developing evaluation metrics that account for this reliability–informativeness tradeoff is an open challenge. Further, the analysis of drivers of drug response and uncertainty we conduct does not establish causality. The Shapley values here reflect contributions from the trained model, not necessarily biological effects. High attribution scores can arise via correlations with confounding factors such as tissue or cell line identity, rather than mechanistically relevant genes. Future work could identify drivers that are independent of cell line or tissue confounders, for example, by applying tissue-invariant risk minimization that controls for tissue or cell line context. The stability of the uncertainty drivers across independent datasets remains to be established.

Despite these limitations, we recommend uncertainty estimation as standard practice in drug response modeling. Our results show that uncertainty estimates enable qualitatively new analyses, from identifying molecular drivers of unpredictability to prioritizing experiments under resource constraints. As drug response models move toward more complex and increasingly multimodal architectures, systematic evaluation of uncertainty quality in addition to predictive accuracy will be important for building models that are more transparent about their limits.

## Supplementary Material

vbag246_Supplementary_Data

## Data Availability

The GDSC data are publicly available at https://www.cancerrxgene.org/. The CTRPv2 drug response data are available through the Cancer Therapeutics Response Portal (https://portals.broadinstitute.org/ctrp.v2/). The CCLE RNA-seq gene expression data are available from the Broad Institute CCLE (https://sites.broadinstitute.org/ccle/datasets). The BeatAML *ex vivo* drug response and RNA-seq gene expression data are available through the Beat AML data viewer (http://www.vizome.org/). The code, cross-validation splits, processed input data, and model outputs are available at https://github.com/PascalIversen/LUDRP and Zenodo (https://doi.org/10.5281/zenodo.19219090). For the cross-dataset experiments, we use the preprocessed datasets from DrEval (https://doi.org/10.5281/zenodo.12633909).

## References

[vbag246-B1] Abdar M, Pourpanah F, Hussain S et al A review of uncertainty quantification in deep learning: techniques, applications and challenges. Inform Fusion 2021;76:243–97.

[vbag246-B2] Adam G , Rampášek L, Safikhani Z et al Machine learning approaches to drug response prediction: challenges and recent progress. NPJ Precis Oncol 2020;4:19. 10.1038/s41698-020-0122-1 PMC7296033 32566759

[vbag246-B3] Amini A , Schwarting W, Soleimany A et al Deep evidential regression. In: *Advances in Neural Information Processing Systems*, Vol. 33, p. 14927–37. Red Hook, NY: Curran Associates, Inc, 2020.

[vbag246-B4] Barretina J , Caponigro G, Stransky N et al The Cancer Cell Line Encyclopedia enables predictive modelling of anticancer drug sensitivity. Nature 2012;483:603–7. 10.1038/nature11003 PMC3320027 22460905

[vbag246-B5] Bernett J , Iversen P, Picciani M et al Critical evaluation of drug response prediction models with DrEval. Nat Commun 2026;17:4238. 10.1038/s41467-026-72903-w PMC13168506 42120410

[vbag246-B6] Bishop CM. *Mixture density networks.* Neural Computing Research Group Report NCRG/94/004. Birmingham: Aston University, 1994.

[vbag246-B7] Chakraborti T , Banerji CRS, Marandon A et al Personalized uncertainty quantification in artificial intelligence. Nat Mach Intell 2025;7:522–30.

[vbag246-B8] Chung Y, Char I, Guo H et al Uncertainty Toolbox: an open-source library for assessing, visualizing, and improving uncertainty quantification. arXiv, arXiv:2109.10254, 2021, preprint: not peer reviewed.

[vbag246-B9] Corrales E , Levit-Zerdoun E, Metzger P et al PI3K/AKT signaling allows for MAPK/ERK pathway independency mediating dedifferentiation-driven treatment resistance in melanoma. Cell Commun Signal 2022;20:187. 10.1186/s12964-022-00989-y PMC9700886 36434616

[vbag246-B10] Fakour F , Mosleh A, Ramezani R. A structured review of literature on uncertainty in machine learning & deep learning. arXiv, arXiv:2406.00332, 2024, preprint: not peer reviewed.

[vbag246-B11] Fang Y , Xu P, Yang J et al A quantile regression forest based method to predict drug response and assess prediction reliability. PLoS One 2018;13:e0205155. 10.1371/journal.pone.0205155 PMC6173405 30289891

[vbag246-B12] Gal Y , Ghahramani Z. Dropout as a Bayesian Approximation: Representing Model Uncertainty in Deep Learning. In: *Proceedings of the 33rd International Conference on Machine Learning*, p. 1050–1059. New York, NY: PMLR, 2016.

[vbag246-B13] Ghandi M , Huang FW, Jané-Valbuena J et al Next-generation characterization of the cancer cell line encyclopedia. Nature 2019;569:503–8. 10.1038/s41586-019-1186-3 PMC6697103 31068700

[vbag246-B14] Gillet J-P , Varma S, Gottesman MM. The clinical relevance of cancer cell lines. J Natl Cancer Inst 2013;105:452–8. 10.1093/jnci/djt007 PMC3691946 23434901

[vbag246-B15] Goodfellow IJ , Shlens J, Szegedy C. Explaining and harnessing adversarial examples. In: *3rd International Conference on Learning Representations (ICLR)*, San Diego, CA, USA, 2015.

[vbag246-B16] Greenman KP , Amini AP, Yang KK. Benchmarking uncertainty quantification for protein engineering. PLoS Comput Biol 2025;21:e1012639. 10.1371/journal.pcbi.1012639 PMC11741572 39775201

[vbag246-B17] Gruber C , Schenk PO, Schierholz M et al Sources of uncertainty in supervised machine learning – a statisticians’ view. arXiv, arXiv:2305.16703, 2025, preprint: not peer reviewed.

[vbag246-B18] Haibe-Kains B , El-Hachem N, Birkbak NJ et al Inconsistency in large pharmacogenomic studies. Nature 2013;504:389–93. 10.1038/nature12831 PMC4237165 24284626

[vbag246-B19] Hirschfeld L , Swanson K, Yang K et al Uncertainty quantification using neural networks for molecular property prediction. J Chem Inf Model 2020;60:3770–80. 10.1021/acs.jcim.0c00502 32702986

[vbag246-B20] Hu W , Wartmann T, Strecker M et al Transient receptor potential channels as predictive marker and potential indicator of chemoresistance in colon cancer. Oncol Res 2023;32:227–39. 10.32604/or.2023.043053 PMC10767253 38188686

[vbag246-B21] Iversen P , Witzke S, Baum K et al Identifying Drivers of Predictive Aleatoric Uncertainty. In: *Proceedings of the Thirty-Fourth International Joint Conference on Artificial Intelligence*, Montreal, Canada, p. 5453–62. International Joint Conferences on Artificial Intelligence Organization, 2025.

[vbag246-B22] Kendall A , Gal Y. What uncertainties do we need in Bayesian deep learning for computer vision? In: *Advances in Neural Information Processing Systems*, Vol. 30. Red Hook, NY: Curran Associates, Inc. 2017.

[vbag246-B23] Koenker R , Bassett G. Regression quantiles. Econometrica 1978;46:33–50.

[vbag246-B24] Kuenzi BM , Park J, Fong SH et al Predicting drug response and synergy using a deep learning model of human cancer cells. Cancer Cell 2020;38:672–84.e6. 10.1016/j.ccell.2020.09.014 PMC7737474 33096023

[vbag246-B25] Lakshminarayanan B , Pritzel A, Blundell C. Simple and scalable predictive uncertainty estimation using deep ensembles. In: *Advances in Neural Information Processing Systems*, Vol. 30. Red Hook, NY: Curran Associates, Inc, 2017.

[vbag246-B26] Lee BKB , Tiong KH, Chang JK et al DeSigN: connecting gene expression with therapeutics for drug repurposing and development. BMC Genomics 2017;18:934. 10.1186/s12864-016-3260-7 PMC5310278 28198666

[vbag246-B27] Lenhof K , Eckhart L, Rolli L-M et al Trust me if you can: a survey on reliability and interpretability of machine learning approaches for drug sensitivity prediction in cancer. Brief Bioinform 2024a;25:bbae379. 10.1093/bib/bbae379 PMC11299037 39101498

[vbag246-B28] Lenhof K , Eckhart L, Rolli L-M et al Reliable anti-cancer drug sensitivity prediction and prioritization. Sci Rep 2024b;14:12303. 10.1038/s41598-024-62956-6 PMC11137046 38811639

[vbag246-B29] MacKay DJC. Bayesian interpolation. Neural Comput 1992;4:415–47.

[vbag246-B30] Maeda T , Suzuki A, Koga K et al TRPM5 mediates acidic extracellular pH signaling and TRPM5 inhibition reduces spontaneous metastasis in mouse B16-BL6 melanoma cells. Oncotarget 2017;8:78312–26. 10.18632/oncotarget.20826 PMC5667964 29108231

[vbag246-B31] Manica M , Oskooei A, Born J et al Toward explainable anticancer compound sensitivity prediction via multimodal attention-based convolutional encoders. Mol Pharm 2019;16:4797–806. 10.1021/acs.molpharmaceut.9b00520 31618586

[vbag246-B32] Mentch L , Hooker G. Quantifying uncertainty in random forests via confidence intervals and hypothesis tests. J Mach Learn Res 2016;17:1–41.

[vbag246-B33] Nikeghbal P , Zamanian D, Burke D et al Organoid models established from primary tumors and patient-derived xenograft tumors reflect platinum sensitivity of ovarian cancer patients. BMC Cancer 2025;25:1459. 10.1186/s12885-025-14811-8 PMC12482882 41029529

[vbag246-B34] Nix DA, Weigend AS. Estimating the mean and variance of the target probability distribution. In: *Proceedings of 1994 IEEE International Conference on Neural Networks (ICNN’94)*, Vol. 1, p. 55–60. Piscataway, NJ: IEEE, 1994.

[vbag246-B35] Nolte D , Ghosh S, Pal R. Efficient normalized conformal prediction and uncertainty quantification for anti-cancer drug sensitivity prediction with deep regression forests. In: *2024 46th Annual International Conference of the IEEE Engineering in Medicine and Biology Society (EMBC)*, p. 1–6. Piscataway, NJ: IEEE, 2024. 10.1109/EMBC53108.2024.10782378 40039751

[vbag246-B36] Obermeyer Z , Powers B, Vogeli C et al Dissecting racial bias in an algorithm used to manage the health of populations. Science 2019;366:447–53. 10.1126/science.aax2342 31649194

[vbag246-B37] O’Connor OA , Tobinai K. Putting the clinical and biological heterogeneity of non-Hodgkin lymphoma into context. Clin Cancer Res 2014;20:5173–81. 10.1158/1078-0432.CCR-14-0574 25320367

[vbag246-B38] Padilla OHM , Tansey W, Chen Y. Quantile regression with ReLU networks: estimators and minimax rates. J Mach Learn Res 2022;23:1–42.

[vbag246-B39] Peres da Silva R , Suphavilai C, Nagarajan N. TUGDA: task uncertainty guided domain adaptation for robust generalization of cancer drug response prediction from in vitro to in vivo settings. Bioinformatics 2021;37:i76–83. 10.1093/bioinformatics/btab299 PMC8275325 34000002

[vbag246-B40] Prasse P , Iversen P, Lienhard M et al Pre-training on in vitro and fine-tuning on patient-derived data improves deep neural networks for anti-cancer drug-sensitivity prediction. Cancers (Basel) 2022;14:3950. 10.3390/cancers14163950 PMC9406038 36010942

[vbag246-B41] Raghavan S. How inclusive are cell lines in preclinical engineered cancer models? Dis Model Mech 2022;15:dmm049520. 10.1242/dmm.049520 PMC9187871 35642685

[vbag246-B42] Rasmussen MH , Duan C, Kulik HJ et al Uncertain of uncertainties? A comparison of uncertainty quantification metrics for chemical data sets. J Cheminform 2023;15:121. 10.1186/s13321-023-00790-0 PMC10729461 38111020

[vbag246-B43] Rogers D , Hahn M. Extended-connectivity fingerprints. J Chem Inf Model 2010;50:742–54. 10.1021/ci100050t 20426451

[vbag246-B44] Rolli L-M , Eckhart L, Herrmann L et al Increasing trustworthiness of machine learning-based drug sensitivity prediction with a multivariate random forest approach. Digit Discov 2026;5:1746–64.

[vbag246-B45] Seashore-Ludlow B , Rees MG, Cheah JH et al Harnessing connectivity in a large-scale small-molecule sensitivity dataset. Cancer Discov 2015;5:1210–23. 10.1158/2159-8290.CD-15-0235 PMC4631646 26482930

[vbag246-B46] Shi H , Xu T, Li X et al DRExplainer: quantifiable interpretability in drug response prediction with directed graph convolutional network. Artif Intell Med 2025;163:103101. 10.1016/j.artmed.2025.103101 40056540

[vbag246-B47] Tang Y-C , Gottlieb A. Explainable drug sensitivity prediction through cancer pathway enrichment. Sci Rep 2021;11:3128. 10.1038/s41598-021-82612-7 PMC7862690 33542382

[vbag246-B48] Tibshirani RJ , Foygel Barber R, Candes E et al Conformal prediction under covariate shift. In: *Advances in Neural Information Processing Systems*, Vol. 32. Red Hook, NY: Curran Associates, Inc. 2019.

[vbag246-B49] Tyner JW , Tognon CE, Bottomly D et al Functional genomic landscape of acute myeloid leukaemia. Nature 2018;562:526–31. 10.1038/s41586-018-0623-z PMC6280667 30333627

[vbag246-B50] Wong A , Otles E, Donnelly JP et al External validation of a widely implemented proprietary sepsis prediction model in hospitalized patients. JAMA Intern Med 2021;181:1065–70. 10.1001/jamainternmed.2021.2626 PMC8218233 34152373

[vbag246-B51] Yang W , Soares J, Greninger P et al Genomics of Drug Sensitivity in Cancer (GDSC): a resource for therapeutic biomarker discovery in cancer cells. Nucleic Acids Res 2013;41:D955–61. 10.1093/nar/gks1111 PMC3531057 23180760

[vbag246-B52] Zech JR , Badgeley MA, Liu M et al Variable generalization performance of a deep learning model to detect pneumonia in chest radiographs: a cross-sectional study. PLoS Med 2018;15:e1002683. 10.1371/journal.pmed.1002683 PMC6219764 30399157

