# Supplementary material for: Leveraging uncertainty estimates for drug response prediction in cancer cell lines
Source: Bioinform Adv. 2026 Aug 25;6(1):vbag246. doi: 10.1093/bioadv/vbag246 (PMC13600678; doi:10.1093/bioadv/vbag246)
Supplement: Supplementary file 1 [file vbag246_supplementary_data.pdf]

Supplementary Material

Leveraging Uncertainty Estimates for Drug Response Prediction in Cancer Cell Lines

Pascal Iversen[1,2,*], Bernhard Y Renard[2,3], Katharina Baum[1,2,3]

[1] *Freie Universität Berlin, Department of Mathematics and Computer Science, Berlin, Germany*
[2] *Hasso Plattner Institute, Digital Engineering Faculty, University of Potsdam, Germany*
[3] *Windreich Department of Artificial Intelligence and Human Health & Hasso Plattner Institute for Digital Health at Mount Sinai, Icahn School of Medicine at Mount Sinai, New York, USA*

[*] pascal.iversen@fu-berlin.de

Appendix

Calibration and post-hoc recalibration

Calibration can be trivially improved by recalibration with a post-hoc, monotone mapping on a validation set that preserves the ordering of samples. It also does not consider how accurate the model predictions are overall (a poor model with constant uncertainties can be well calibrated). This motivates us to introduce the area under the uncertainty–reduction curve (AUURC) (smaller is better) as a metric for a joint analysis of the quality of uncertainty estimates and point predictions. The curve is constructed by ranking test instances by predicted uncertainty, and computing the mean squared error (MSE) of the retained subset as the most uncertain instances are iteratively removed. A small area under this curve can arise from uncertainties that rank model error well or low overall point prediction errors. Conversely, larger areas indicate less informative uncertainty estimates or higher overall errors. We approximate the AUURC using numerical integration via the trapezoidal rule. We also evaluate the MSE@k%, i.e., the MSE when predicting only the k% instances with the lowest predicted uncertainty. This metric can be used to estimate the empirical risk if predictions on high-uncertainty instances are deferred. We use uncertainty-based deferral as an evaluation scenario for the informativeness of predicted uncertainties, not as a proposed decision-making strategy. In practice, uncertainty would complement domain knowledge in selecting candidates for further investigation.

Sensitivity to the Gaussian approximation

Calibration and sharpness require prediction intervals, and we obtain these by treating each predicted uncertainty as the standard deviation of a Gaussian. For models whose predictive distribution is not parametric, this could in principle distort the comparison. We therefore recompute the Random Forest (RF) coverage non-parametrically: for each test point we treat the predictions of the 500 individual trees as an empirical predictive sample and measure the fraction of true responses falling inside its central inter-quantile ranges, then compare this with the coverage implied by a Gaussian of the same mean and standard deviation. Across the five cross-validation folds the empirical distribution gives a *larger* miscalibration area than the Gaussian approximation (0.157 ± 0.006 versus 0.101 ± 0.006; Figure S1), and both reliability curves lie below the diagonal. The RF is thus overconfident under either treatment, and the Gaussian approximation understates rather than exaggerates its miscalibration.

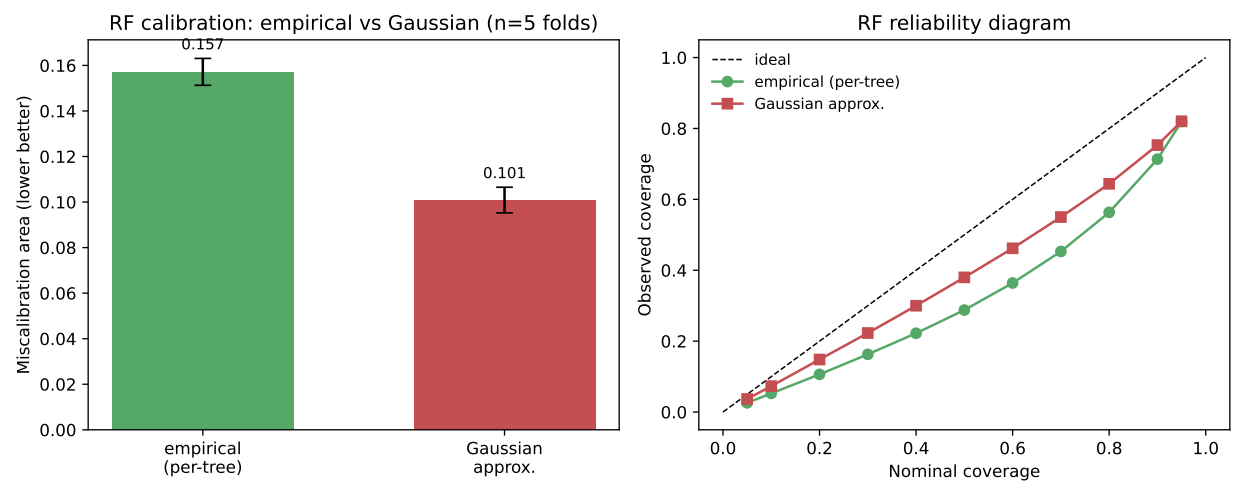

Fig. S1. Sensitivity of the RF calibration to the Gaussian approximation. (a) Miscalibration area computed from the empirical per-tree predictive distribution versus the Gaussian approximation used in the main text (mean $\pm$ standard deviation over the five cross-validation folds; lower is better). (b) Reliability diagram averaged over folds. Both curves lie below the diagonal, i.e. the RF is overconfident under either treatment, and the Gaussian approximation is the more favourable of the two.

Training details

If not mentioned otherwise, experiments are conducted using the Genomics of Drug Sensitivity in Cancer (GDSC) dataset. RNA microarray gene expression features are arcsinh-transformed and z-normalized using train set statistics. Drug features are represented as 128-bit Morgan fingerprints. The final input vector is obtained by concatenating gene expression and drug features. The target variable is the logarithmic z-scaled IC50.

Model training and evaluation follows a five-fold cross-validation protocol, where we exclude cell lines in the test from the training set. Within each fold, a leave-cell-line-out validation set is split from the training set for inner hold-out hyperparameter optimization with exhaustive grid search. The models are fitted on the full train set with the hyperparameter that yielded the lowest-MSE after hyperparameter optimization. Model performance metrics are then estimated on the test set.

For neural network models (Quantile Neural Network (QuantileNN), Monte Carlo Dropout Neural Network (MCDropout), Gaussian Neural Network (GaussNN), Gaussian Neural Network Ensemble (GaussNNEns)), we additionally split off a leave-cell-line-out early-stopping-validation set from each training set. We set a maximum of 1000 Epochs and stop the training when the early-stopping-validation loss ceases to improve for five epochs (patience parameter), and restore the best model with respect to the early-stopping-validation loss after the training stops. We implement all neural models in PyTorch, use ReLU activation, and train with the Adam optimizer with default parameters and a batch size of 128. The ensemble model (GaussNNEns) uses fixed architectures of $[128, 32, 16]$ with dropout 0.3 and 10 ensemble members to reduce computational cost. For the CTRPv2 replication and cross-dataset, we use 5 ensemble members to reduce the computational cost. For all other neural network models, we conduct hyperparameter optimization over network architectures with hidden layer configurations of $[64, 16, 8]$, $[128, 32, 16]$, and $[64, 16, 16, 8, 8]$ units, combined with dropout rates of 0.1 and 0.3. For the Evidential Deep Learning (EvidentialDL) model, we use the same architecture search space and additionally optimize

the evidence regularization weight over $\lambda \in \{10^{-2}, 10^{-1}\}$. We verified that a wider search ($\lambda \in \{10^{-3}, 10^{-2}, 10^{-1}, 1\}$) does not improve EvidentialDL. The GaussNN and GaussNNEns are trained with adversarial training following the Fast Gradient Sign Method (FGSM) (Goodfellow et al., 2015; Lakshminarayanan et al., 2017). At each training step, an adversarial example is generated by perturbing the input $\mathbf{x}$ as $\mathbf{x}_{\text{adv}} = \mathbf{x} + \epsilon \cdot \text{sign}(\nabla_{\mathbf{x}} \mathcal{L}(\mathbf{x}, y))$, with $\epsilon = 0.01$. The total training loss is the sum of the standard loss and the loss on the adversarial example. Classical machine learning models (Bayesian Ridge Regression (BayesianRidge), RF) were implemented using scikit-learn. We optimize Bayesian Ridge regression over precision parameters $\alpha_1 \in \{10^{-6}, 10^{-5}, 10^{-4}\}$, $\alpha_2 \in \{10^{-6}, 10^{-4}\}$, with fixed $\lambda_1 = \lambda_2 = 10^{-6}$. For the Random Forest, we explore forests of 200 and 500 trees with maximum depths of 10 and 15, each tree trained on a random 85% of the samples and 85% of the features (row and feature subsampling); the best configuration (500 trees, maximum depth 15) is selected per fold. For the CTRPv2 replication, the Random Forest uses this configuration without further tuning. The code, the cross-validation splits, and the input data for all experiments and evaluations are available at `https://github.com/PascalIversen/LUDRP`. Model parameter count and run times are listed in Table S4.

Gaussian NN Ensemble variance decomposition

Let M denote the number of ensemble members and $m \in \{1, \ldots, M\}$ the member index. Each member outputs $(\hat{\mu}_m(\mathbf{x}_{ij}), \hat{\sigma}^2_m(\mathbf{x}_{ij}))$. The ensemble prediction and uncertainty decomposition of the GaussNNEns model are:

$$\hat{\mu}(\mathbf{x}_{ij}) = \frac{1}{M} \sum_{m=1}^{M} \hat{\mu}_m(\mathbf{x}_{ij}), \tag{1}$$

$$\hat{\sigma}^2_{\text{tot}}(\mathbf{x}_{ij}) = \underbrace{\frac{1}{M} \sum_{m=1}^{M} \hat{\sigma}^2_m(\mathbf{x}_{ij})}_{\text{aleatoric}} + \underbrace{\frac{1}{M} \sum_{m=1}^{M} \left(\hat{\mu}_m(\mathbf{x}_{ij}) - \hat{\mu}(\mathbf{x}_{ij})\right)^2}_{\text{epistemic}} . \tag{2}$$

Table S1. MSE and Pearson correlation between model predictions and experimental logarithmic IC50 values on the CTRPv2 dataset (5-fold leave-cell-line-out). For the drug-wise metrics, MSE is the median over drugs; the standard deviation represents the variability across drugs. The global metrics are calculated for the folds, and the standard deviation is the variation over folds. Best values are shown in bold; second-best are underlined.

	Random Forest	Bayesian Ridge	Quantile NN	MC Dropout NN	Gaussian NN	Gaussian NN Ens.	Evidential DL
Drug-wise							
MSE	**0.160** ± 0.487	0.537 ± 0.982	0.277 ± 0.714	0.279 ± 0.634	0.215 ± 0.575	<u>0.198</u> ± 0.545	0.262 ± 1.014
Pearson	**0.391** ± 0.289	0.261 ± 0.189	0.207 ± 0.213	0.236 ± 0.190	0.297 ± 0.217	<u>0.345</u> ± 0.239	0.242 ± 0.205
Global							
MSE	**0.236** ± 0.008	0.677 ± 0.014	0.321 ± 0.030	0.330 ± 0.019	0.276 ± 0.008	<u>0.274</u> ± 0.008	0.324 ± 0.028
Pearson	**0.874** ± 0.003	0.571 ± 0.006	0.832 ± 0.014	0.823 ± 0.009	0.854 ± 0.003	<u>0.863</u> ± 0.004	0.824 ± 0.015

Table S2. Uncertainty evaluation metrics per model on the CTRPv2 dataset (average ± standard deviation over the cross-validation folds). AUURC: area under the uncertainty–reduction curve (lower = better). MSE@k%: MSE on the k% most confident predictions (lower = better). MA: miscalibration area (lower = better calibrated). Ranking skill: normalized risk–reduction skill, independent of overall accuracy (1 = oracle error ordering, 0 = random; higher = better). $r(|\epsilon|, \hat{\sigma})$: Pearson correlation between absolute error and predicted uncertainty (higher = better). Best values are shown in bold, second-best underlined.

	Random Forest	Bayesian Ridge	Quantile NN	MC Dropout NN	Gaussian NN	Gaussian NN Ens.	Evidential DL
AUURC	**0.099** ± 0.004	0.627 ± 0.009	0.179 ± 0.040	0.219 ± 0.026	0.118 ± 0.008	<u>0.111</u> ± 0.005	0.147 ± 0.015
MSE@10%	<u>0.051</u> ± 0.004	0.612 ± 0.043	0.125 ± 0.053	0.173 ± 0.027	0.056 ± 0.009	**0.049** ± 0.002	0.067 ± 0.003
MSE@50%	**0.091** ± 0.004	0.627 ± 0.006	0.169 ± 0.036	0.213 ± 0.027	0.110 ± 0.009	<u>0.102</u> ± 0.006	0.138 ± 0.016
Ranking skill	<u>0.67</u> ± 0.00	0.09 ± 0.03	0.54 ± 0.07	0.39 ± 0.06	0.66 ± 0.02	**0.69** ± 0.01	0.64 ± 0.01
$r(\|\epsilon\|, \hat{\sigma})$	<u>0.49</u> ± 0.01	0.08 ± 0.05	0.40 ± 0.05	0.26 ± 0.07	0.49 ± 0.01	**0.52** ± 0.01	0.42 ± 0.04
MA	**0.01** ± 0.00	0.22 ± 0.00	0.03 ± 0.01	0.31 ± 0.03	<u>0.03</u> ± 0.01	0.04 ± 0.01	0.08 ± 0.04

Table S3. Transfer mean squared error (on the globally z-scored response, smaller is better) for the cross-dataset out-of-distribution scenarios of Figure 3(B), ordered by increasing shift. The GDSC transfers are averaged over five random cell-line splits; the BeatAML transfer uses a single split.

Model	in-dist	CTRPv2→GDSC (harm.)	CTRPv2→BeatAML	CTRPv2→GDSC (native)
Bayesian Ridge	0.69	1.02	1.63	36.44
Random Forest	0.25	0.38	1.16	0.45
MC Dropout	0.32	0.54	1.74	16.90
Quantile NN	0.35	0.62	2.14	8.15
Gaussian NN Ensemble	0.28	0.50	1.60	3.96
Evidential DL	0.34	0.62	1.63	18.64
Gaussian NN	0.30	0.53	1.50	8.73

Table S4. Number of model parameters and computation time for training and test set prediction on one fold. Device: Apple MPS.

Model	# Parameters	Train (s)	Time/Epoch (s)	Predict (s)
Random Forest	—	698.3	—	12.84
Bayesian Ridge	—	64.5	—	3.04
Quantile NN	288,611	94.7	8.61	0.11
MC Dropout NN	288,577	141.9	6.17	0.64
Gaussian NN	288,594	392.7	14.02	0.12
Gaussian NN Ens.	2,885,940	8534.3	—	2.06
Evidential DL	288,628	184.3	10.84	0.12

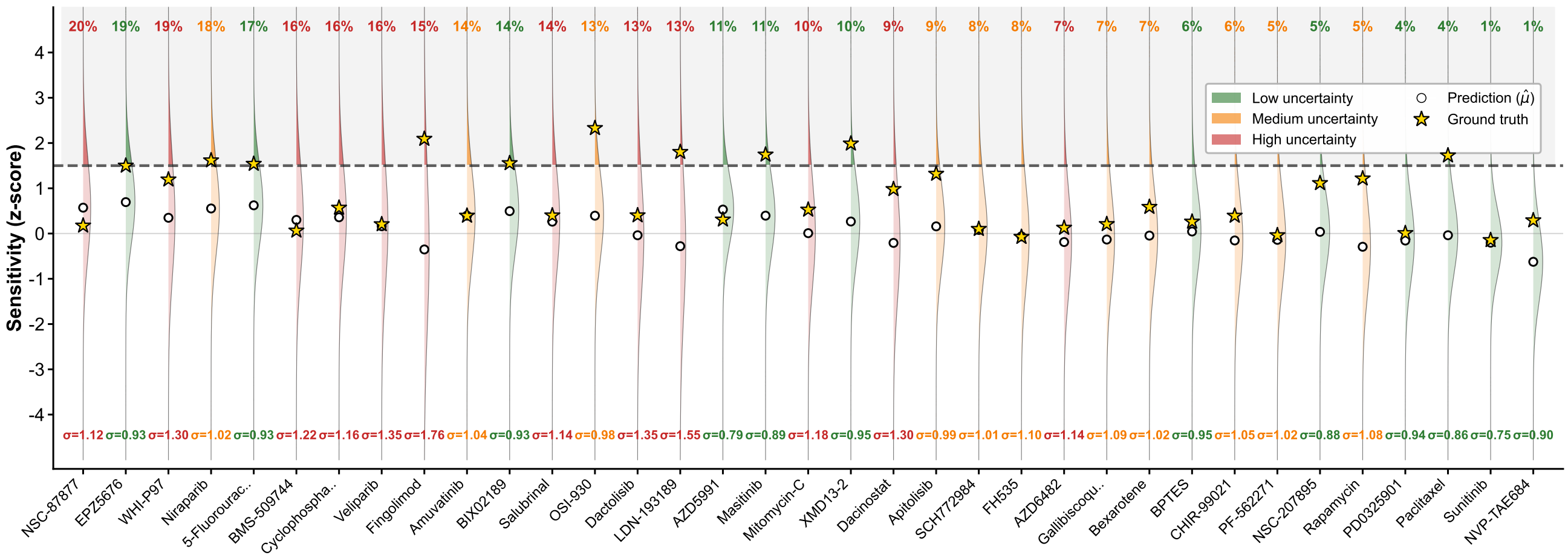

Fig. S2. Predicted and experimental relative drug sensitivity distributions for the CESS leukemia cell line across a sample of 35 drugs. Sensitivities are the per-drug z-scored negative logarithmic IC50. Each drug's predictive distribution of the GaussNNEns on GDSC data is shown. Drugs are sorted by the probability of exceeding a sensitivity threshold of $z = 1.5$, shown as a percentage above each drug.

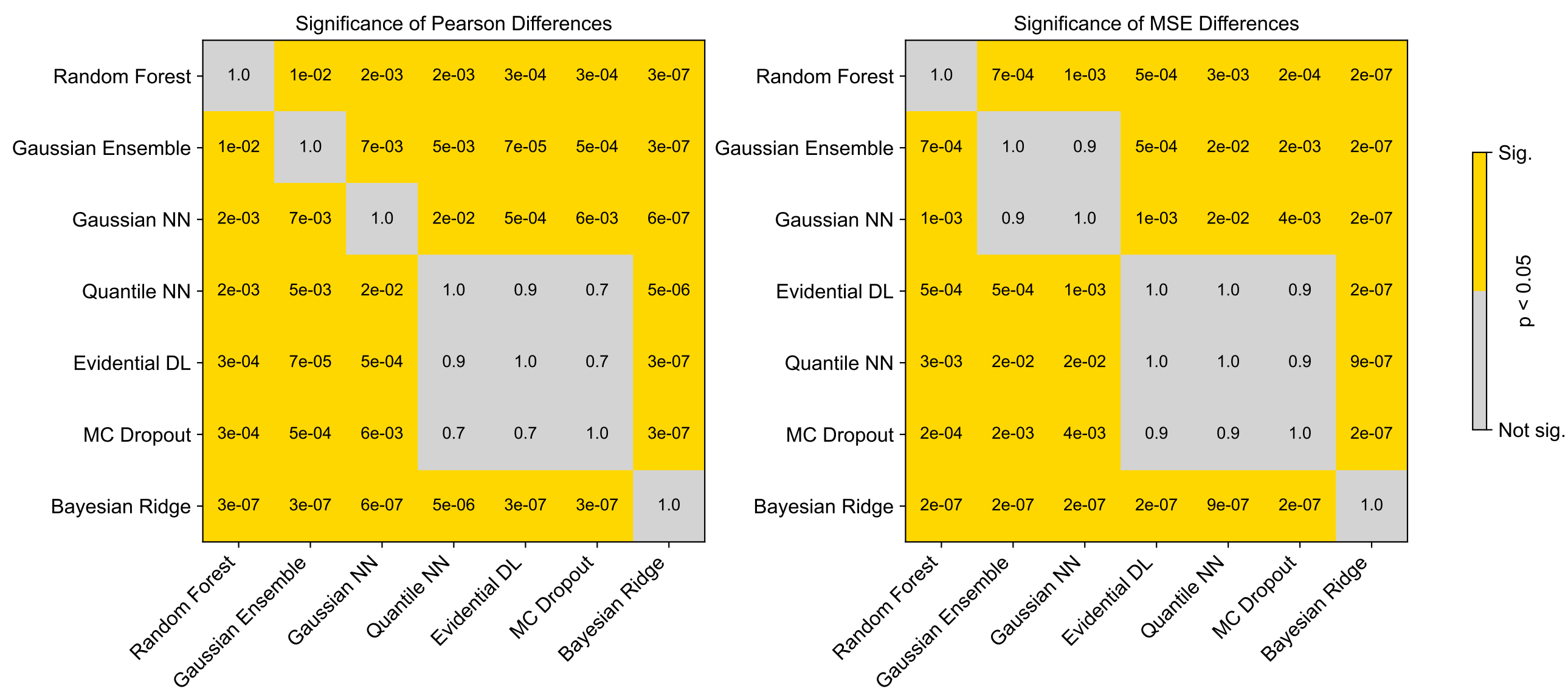

Fig. S3. Pairwise statistical comparison of model accuracy for prediction on GDSC across metrics. Each cell shows the result of a paired two-sided t-test between two models over cross-validation folds, corrected for multiple testing using the Benjamini–Hochberg procedure. Left: significance of differences in global Pearson correlations after Fisher-z transformation. Right: significance of differences in global mean squared error. Models are sorted for performance.

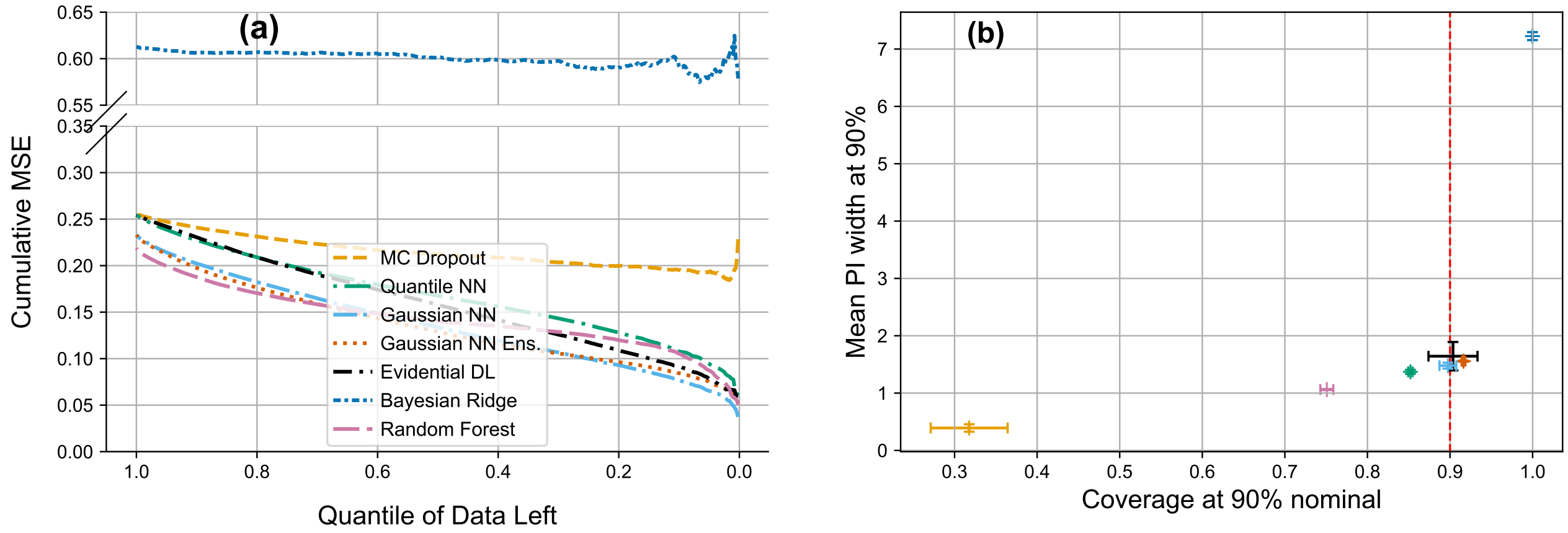

Fig. S4. (a) Uncertainty-based filtering curves for each model on GDSC data, including Bayesian Ridge. The x-axis shows the fraction of test samples retained after thresholding by predicted uncertainty (most uncertain removed first, left to right). The y-axis reports the MSE computed on the reduced test subset. A steeper decline indicates a more effective uncertainty estimation. (b) Calibration–sharpness tradeoff across models. Each point is the mean empirical coverage and average prediction interval (PI) width at 90 % nominal coverage. Error bars indicate variability across folds.

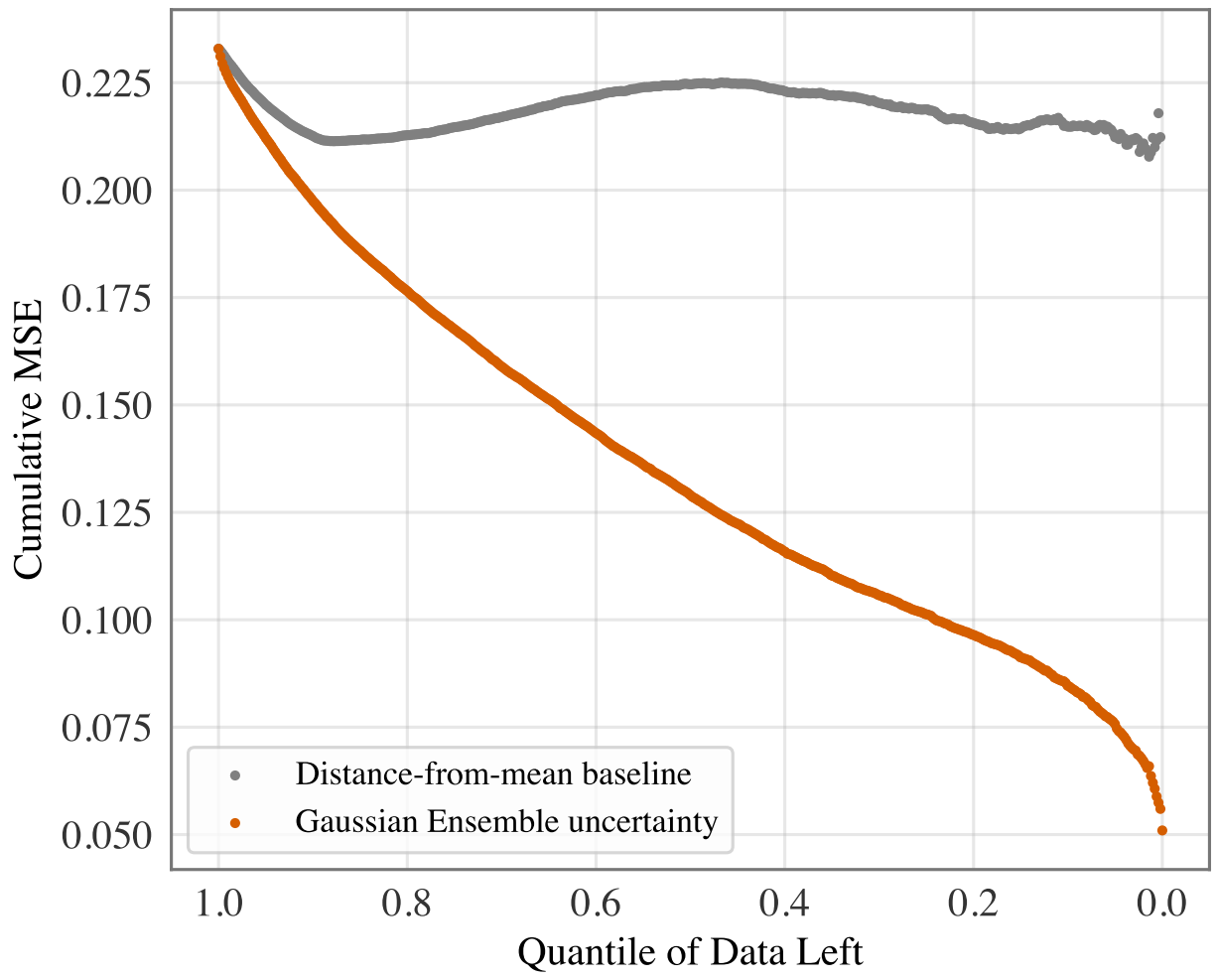

Fig. S5. Uncertainty reduction curve for the GaussNNEns on GDSC compared to a baseline, where instances are ordered by the absolute distance of their predictions from the mean prediction.

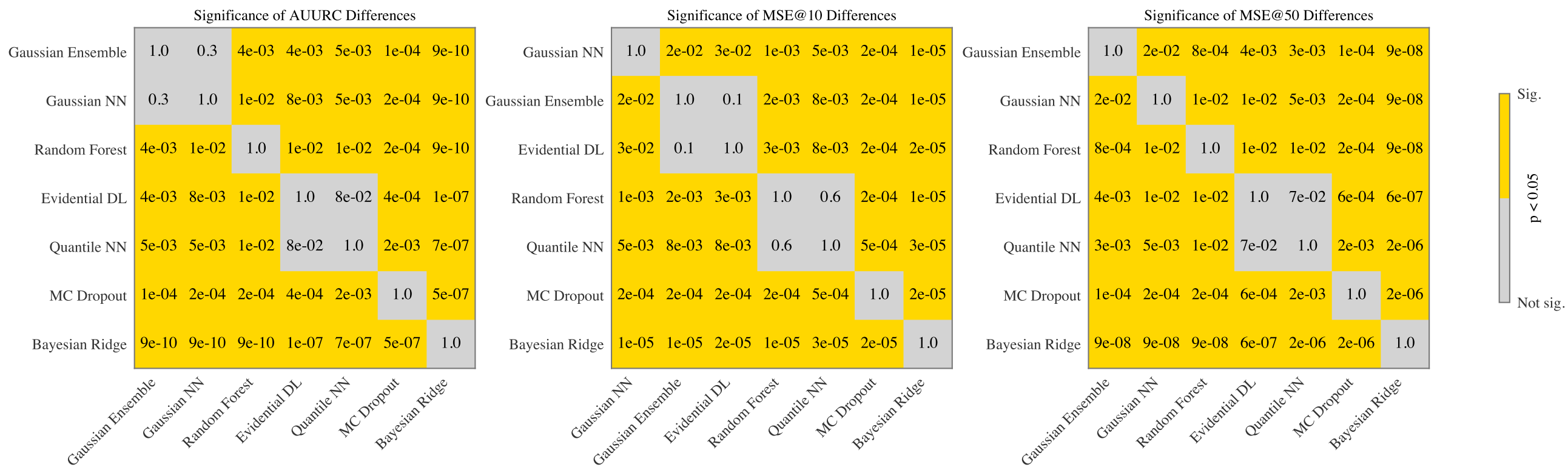

Fig. S6. Pairwise statistical comparison of uncertainty metrics for prediction on GDSC across metrics. Each cell shows the result of a paired two-sided t-test between two models over cross-validation folds, corrected for multiple testing using the Benjamini–Hochberg procedure. Models are sorted for performance.

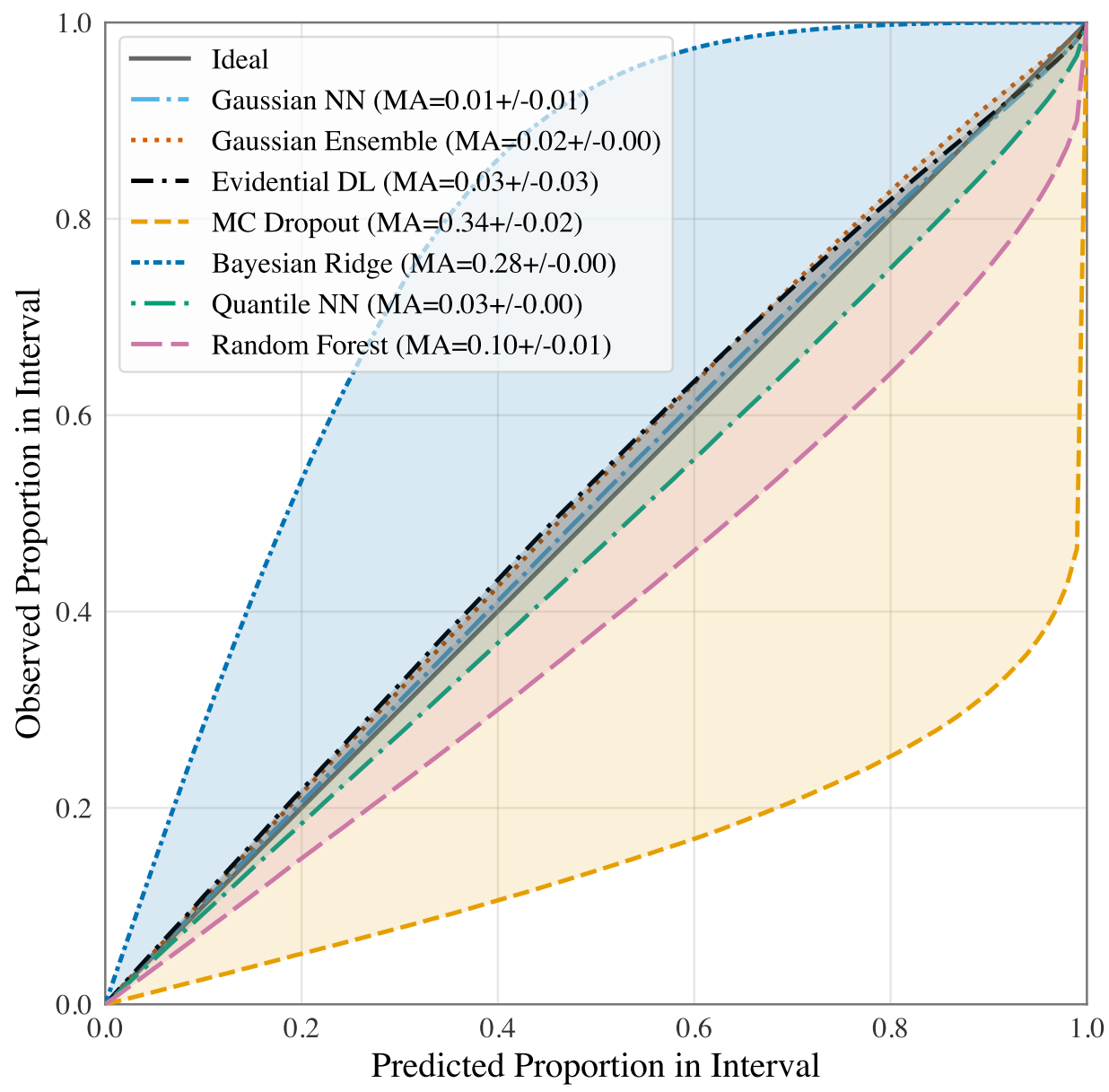

Fig. S7. Calibration curves comparing predicted and observed proportions of responses within uncertainty intervals for each model on GDSC data. Models with curves close to the ideal diagonal are well calibrated (GaussNN, GaussNNEns, EvidentialDL, QuantileNN). Curves above the diagonal indicate underconfident predictions (BayesianRidge), while curves below indicate overconfident predictions (MCDropout, RF). Shaded regions correspond to miscalibration areas (MA) for each model. All calibration results assume a Gaussian predictive distribution.

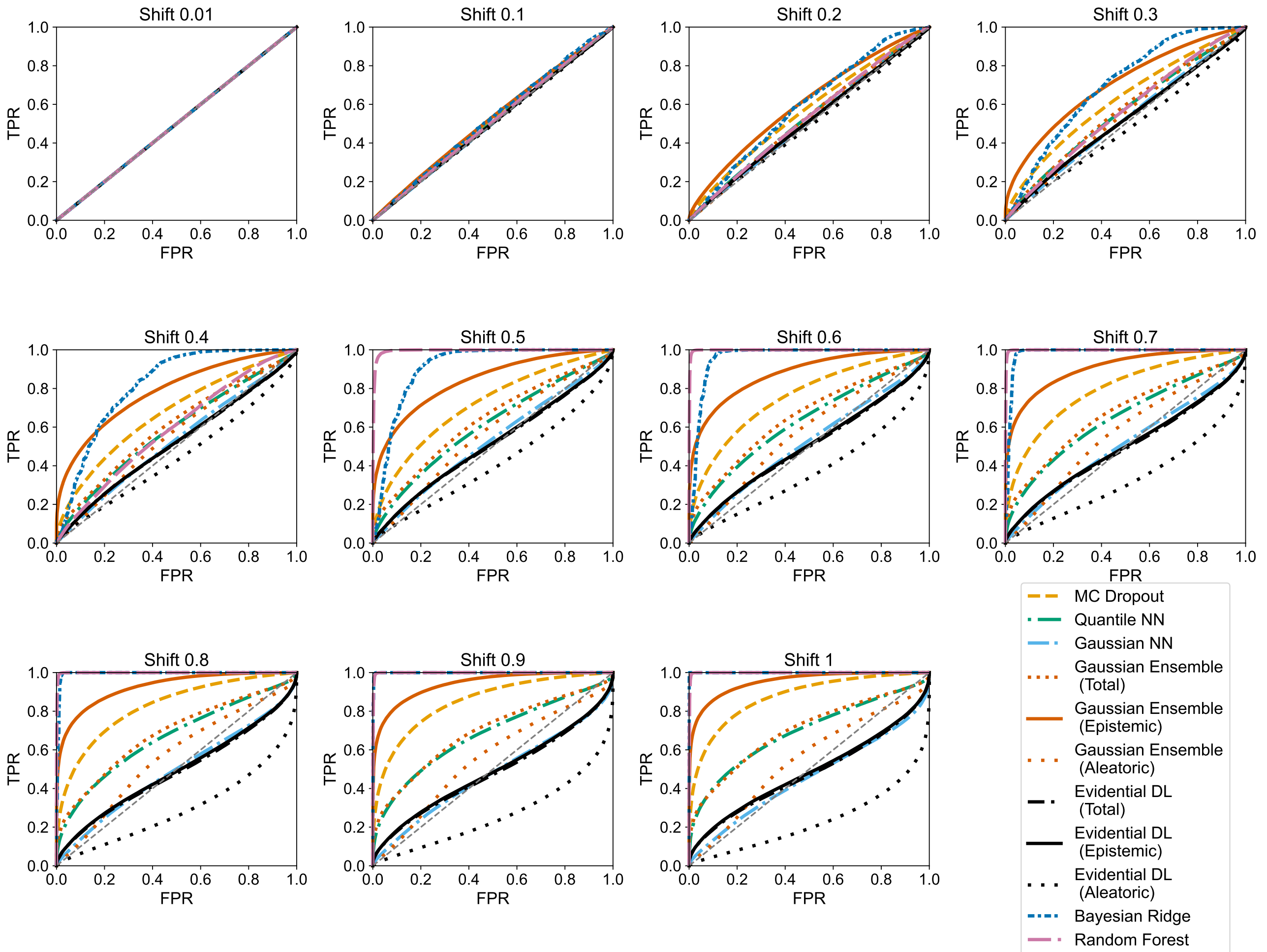

Fig. S8. Receiver Operating Characteristic (ROC) curves for out-of-distribution detection performance on GDSC across different distribution shifts induced by synthetic perturbations. Each subplot corresponds to one shift level, showing true positive rate (TPR) versus false positive rate (FPR). We also show GaussNNEns uncertainty decomposed into epistemic and aleatoric components.

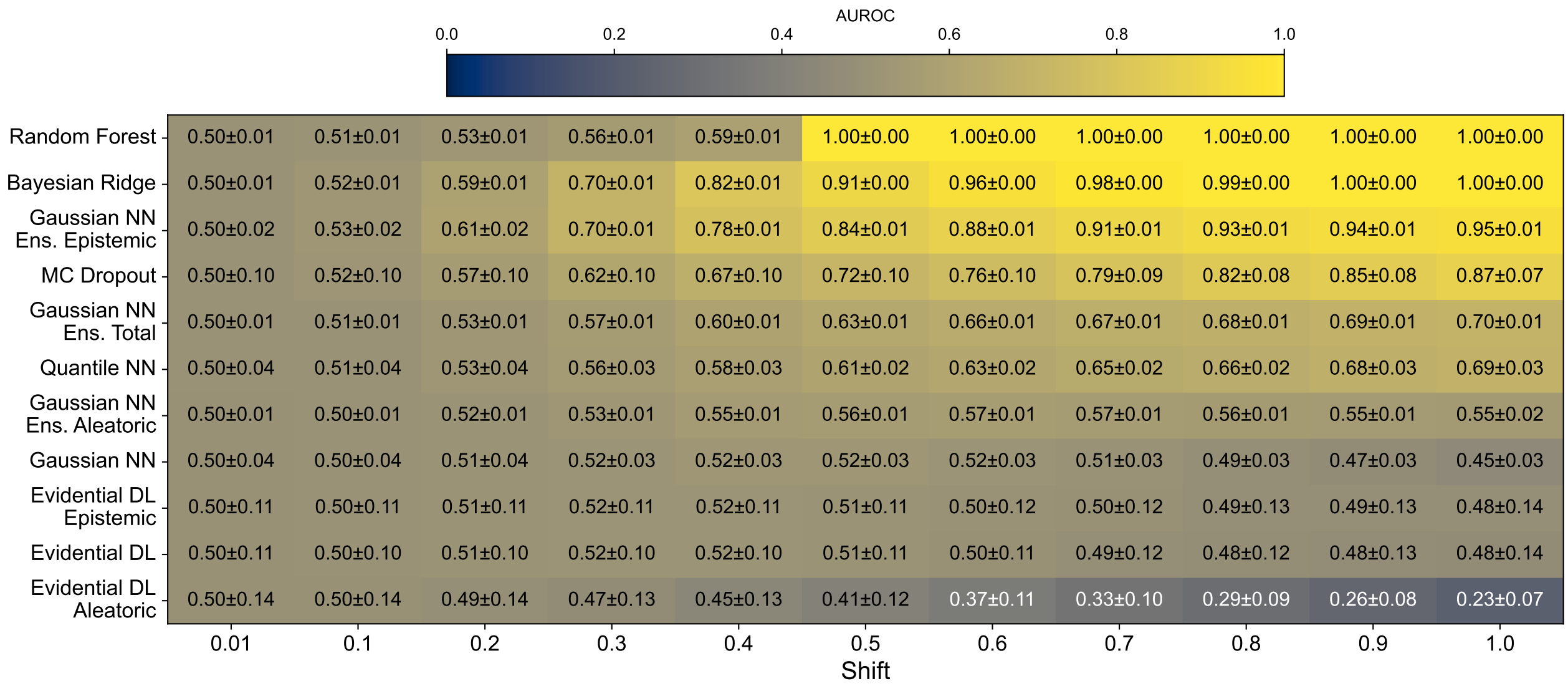

Fig. S9. Out-of-distribution (OOD) detection on GDSC-trained models: area under the receiver operating characteristic curve (higher is better) for distinguishing synthetically perturbed from unperturbed samples based on predicted uncertainty for increasing distribution shift strengths. Variation is the standard deviation of the area under the receiver operating characteristic curve over the five cross-validation folds.

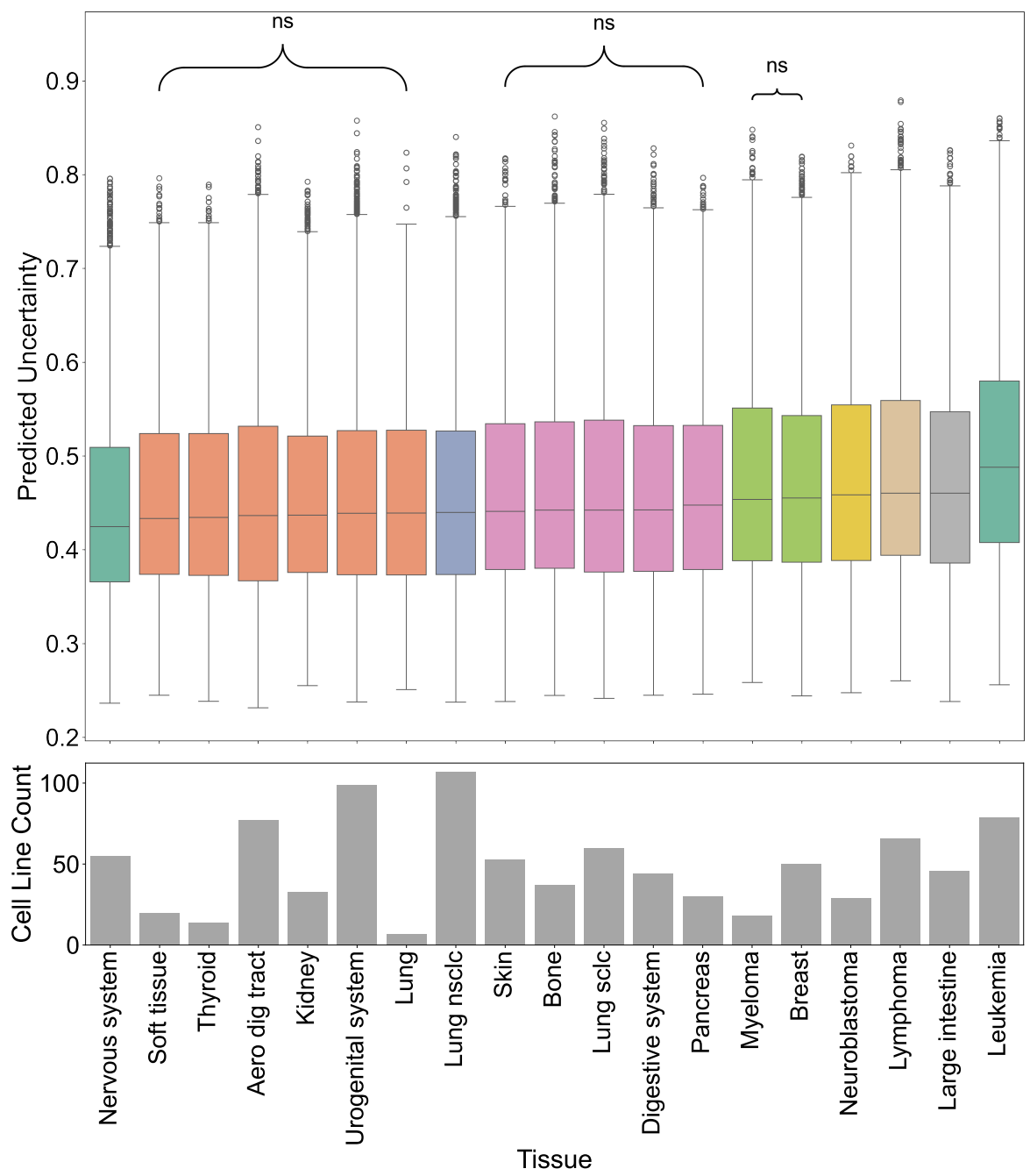

(a) Tissue stratified aleatoric uncertainty

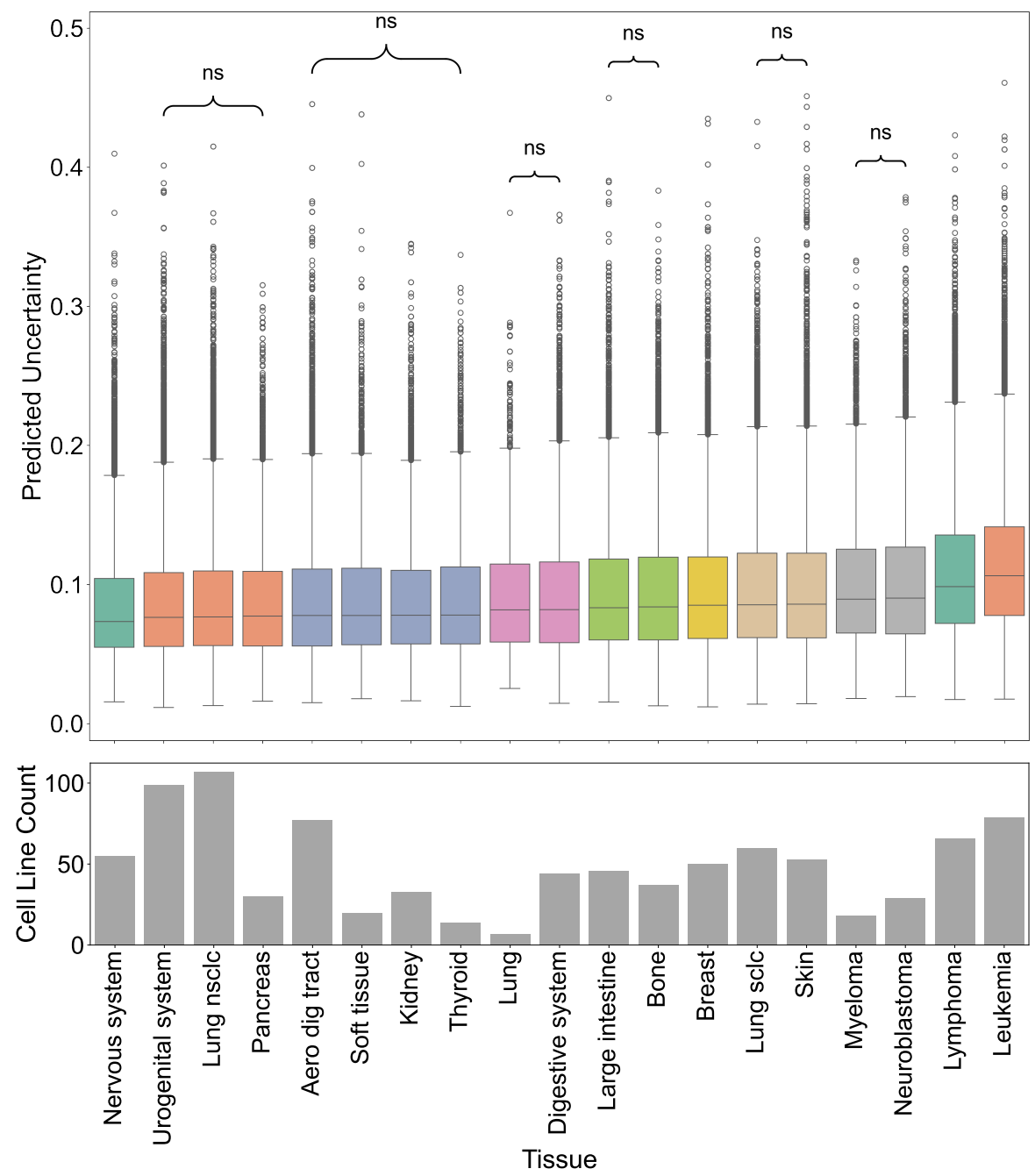

(b) Tissue stratified epistemic uncertainty

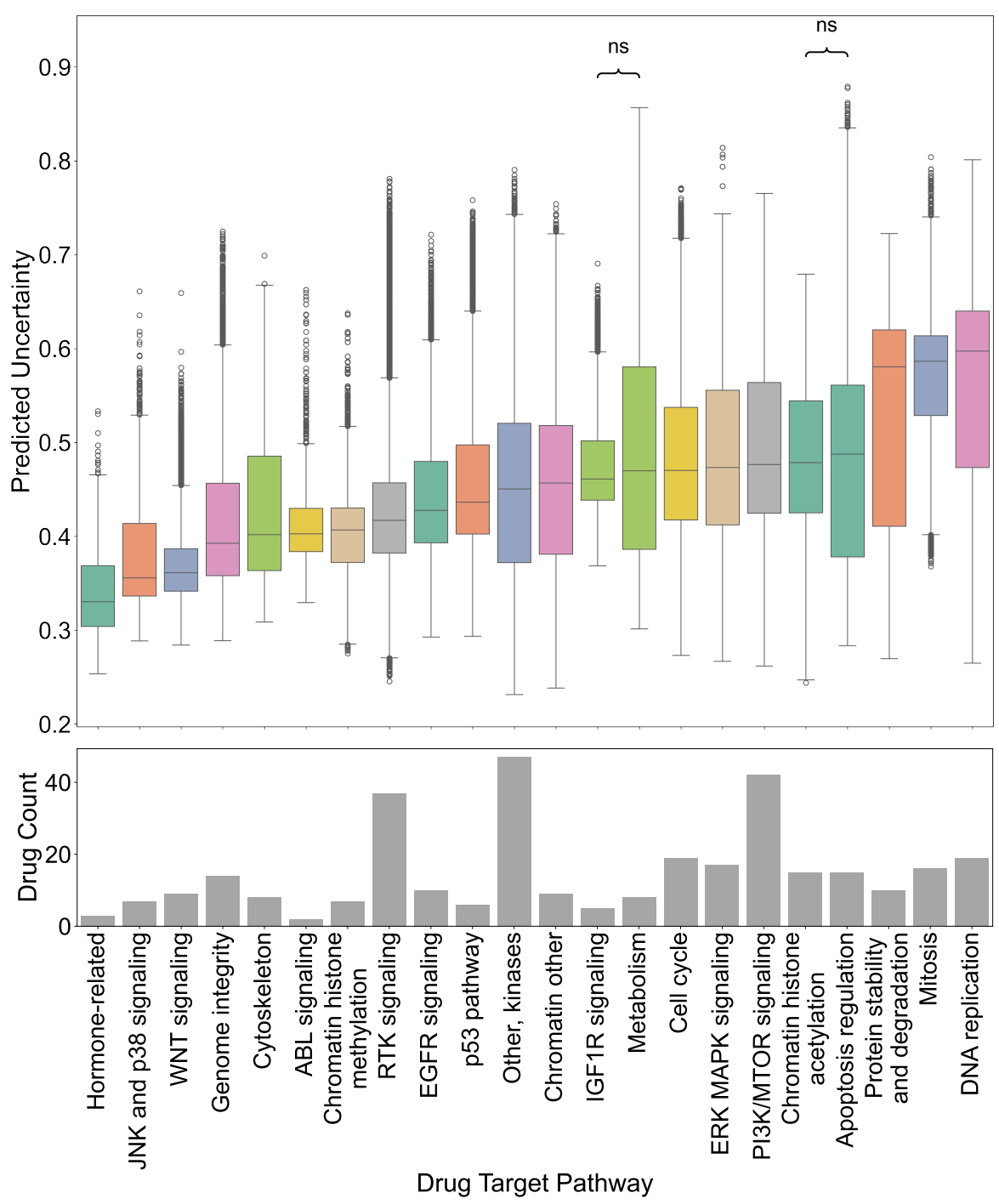

(c) Pathway stratified aleatoric uncertainty

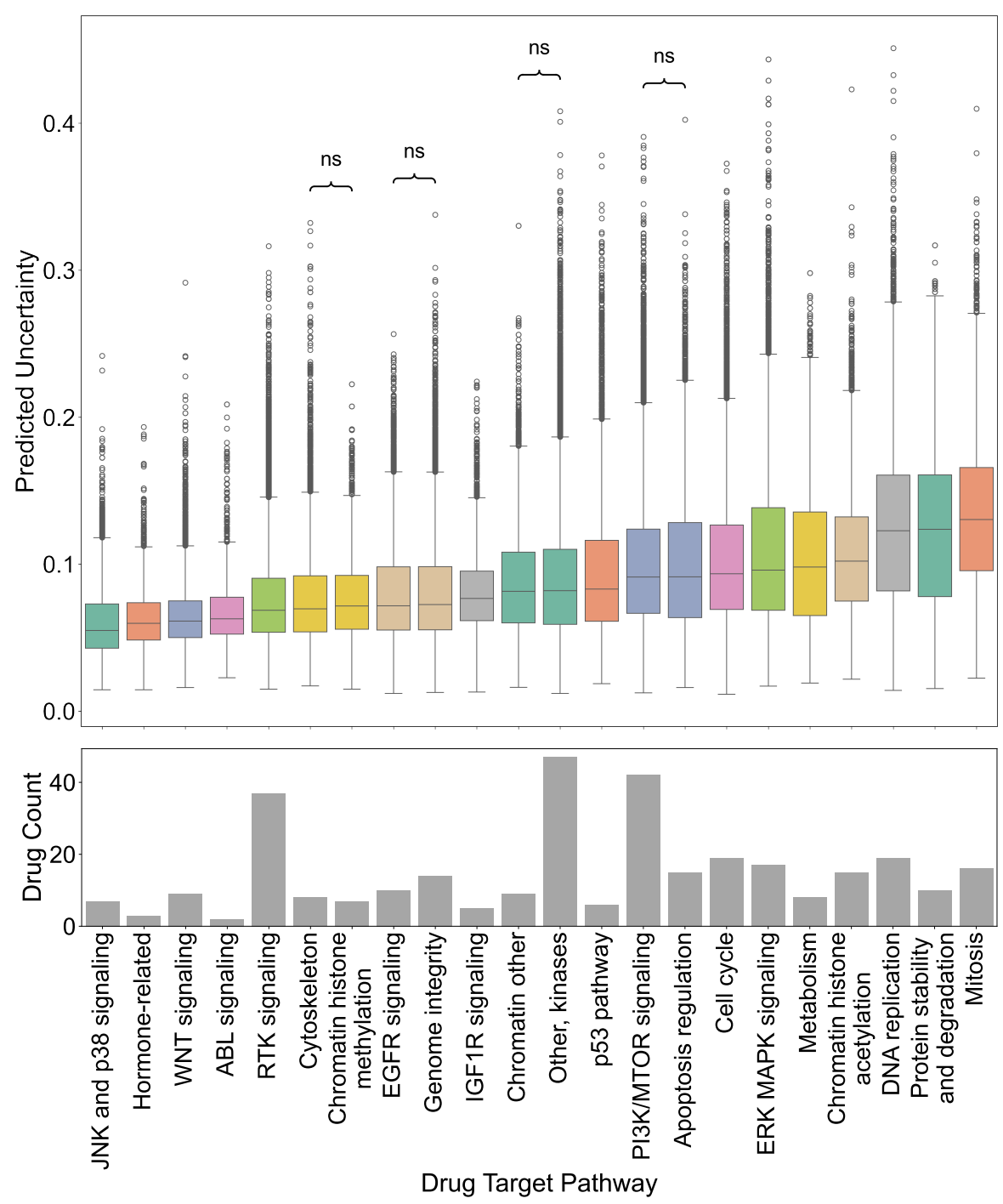

(d) Pathway stratified epistemic uncertainty

Fig. S10. Comparison of predictive uncertainties estimated by the GaussNNEns on GDSC. (a, b) Aleatoric and epistemic uncertainty across tissues. (c, d) Aleatoric and epistemic uncertainty across drug target pathways. Statistical differences between groups were assessed using post hoc pairwise Dunn's test with Holm correction. Non-significant pairwise differences are marked with 'ns'. Lower panels of each subplot show sample counts per group.

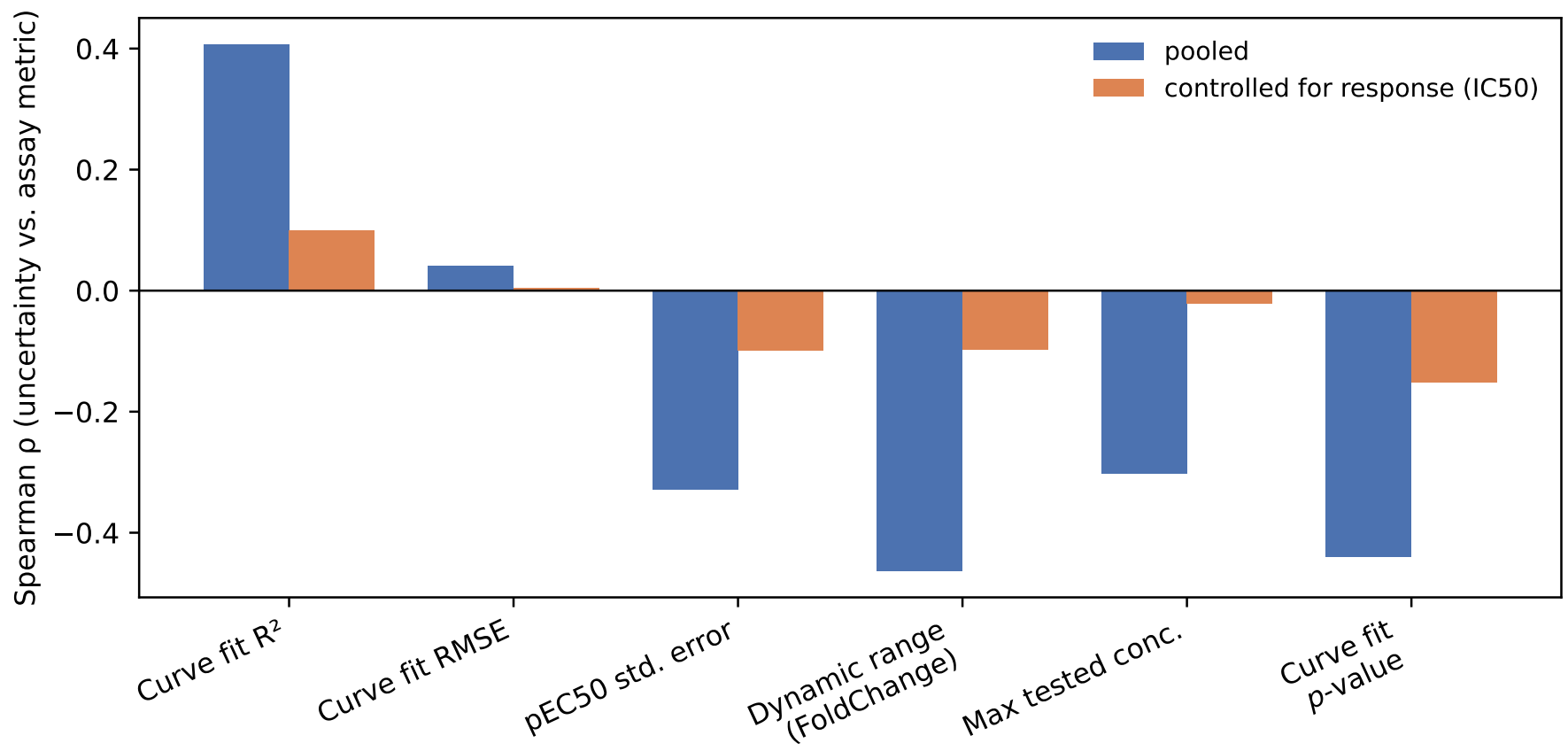

Fig. S11. Predicted aleatoric uncertainty of the GaussNNEns on GDSC versus dose-response assay-quality metrics (CurveCurator re-fit of GDSC), shown as Spearman correlations across all pairs (blue) and after controlling for the measured response value (IC50; first-order partial Spearman, orange). The predicted uncertainty correlates moderately with assay properties such as dynamic range and curve-fit R^2, but these associations are largely mediated by the confounding effect of the overall IC50, because the assay metrics are proxies for the response. The root mean squared error (RMSE) of the curve fit is essentially uncorrelated with uncertainty.

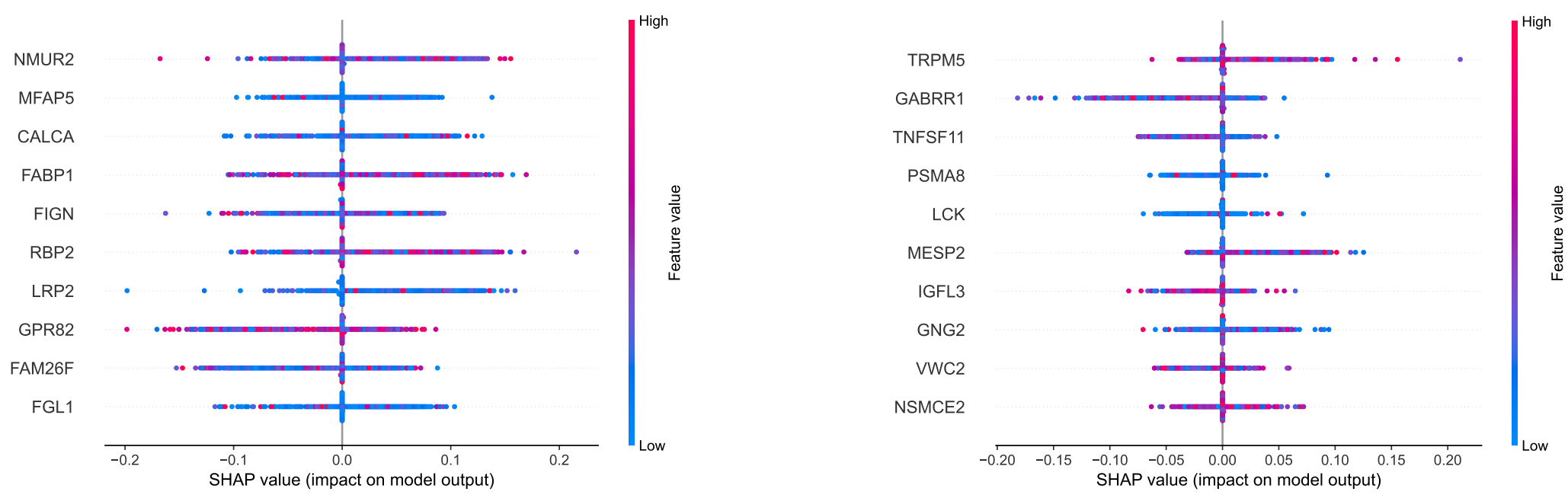

(a) (b)

Fig. S12. Top-10 gene-level contributions to (a) the predicted mean drug response and (b) the predicted uncertainty of the GaussNN for the test set of one cross-validation fold measured as estimated Shapley values on GDSC. Each point corresponds to a drug-cell-line pair sample. Genes are ordered by overall importance. Notably, distinct sets of genes contribute to response prediction versus uncertainty estimation.

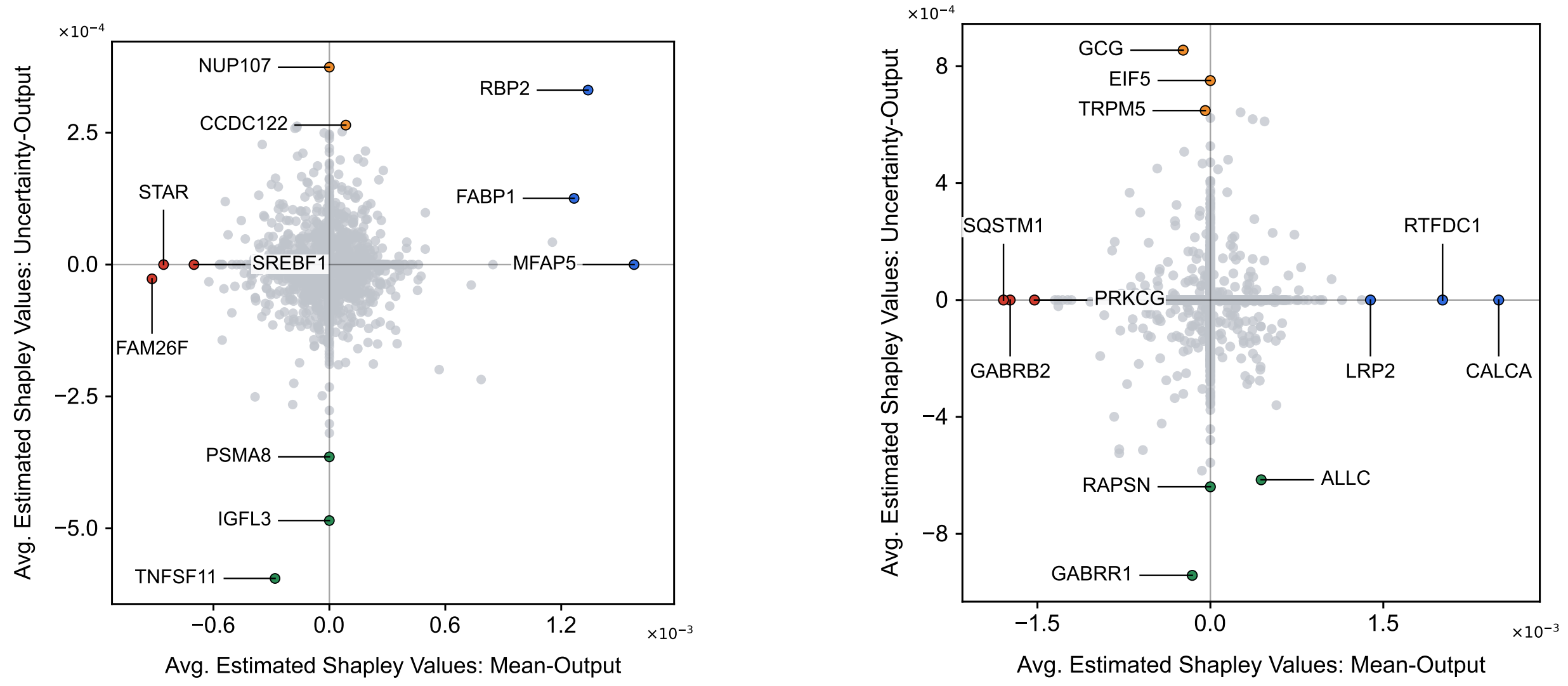

(a) (b)

Fig. S13. Tissue and pathway stratified estimated Shapley values of gene-expression features for predicted mean drug response and predictive uncertainty for the GaussNN on GDSC for (a) Leukemia and (b) nervous system cell lines with respect to ERK-MAPK targeting drugs. The strongest average contributions across the test set, driving weak response, strong response, uncertainty, and certainty are marked in blue, red, orange, and green, respectively.

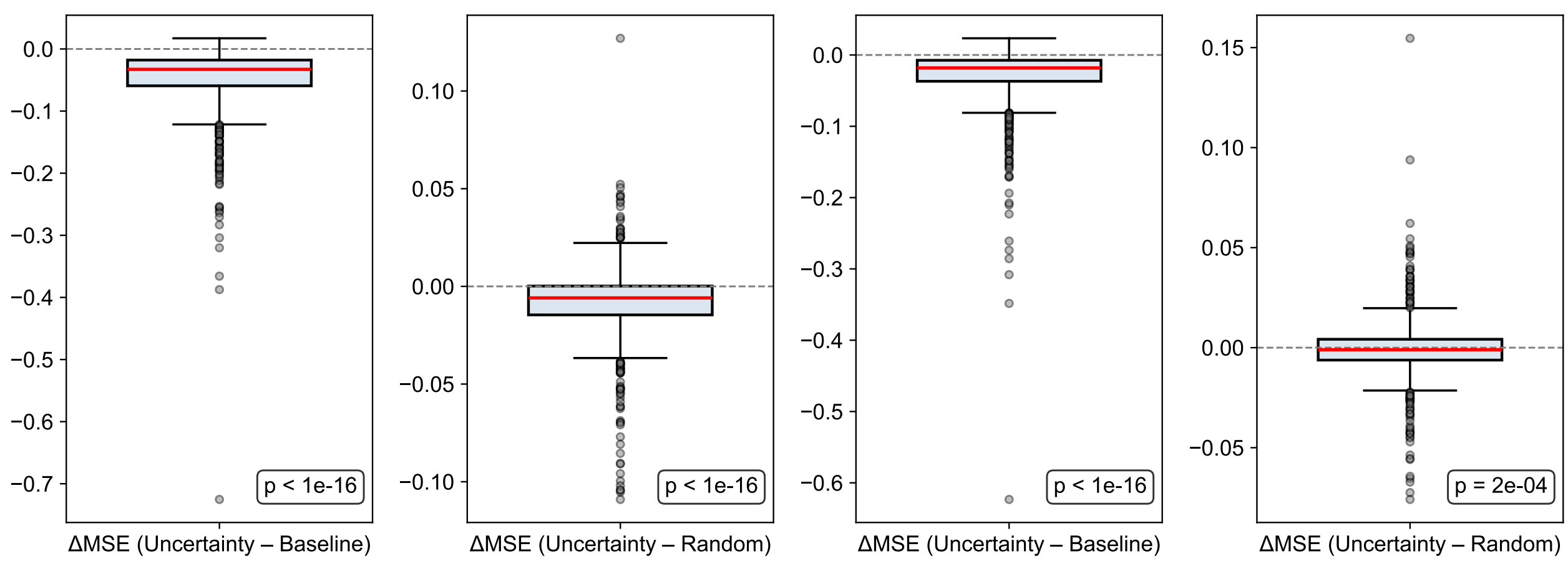

(a) (b) (c) (d)

Fig. S14. Cell-line–wise differences in mean squared error (ΔMSE) between fine-tuning on an uncertainty-guided, personalized selection of drugs and (a, c) the non-fine-tuned baseline model and (b, d) a model fine-tuned on random drugs, all on GDSC. In (a) and (b), the error is averaged over all drugs; in (c) and (d), it is evaluated only on the subset of drugs not included in either fine-tuning strategy. The GaussNN is first trained on a leave-cell-line-out split, then, for each held-out cell line, fine-tuned with either the 60 drugs with the highest predictive uncertainty or a random subset of 60 drugs added to the training set. Significance is assessed using two-sided Wilcoxon tests.

References

I. J. Goodfellow, J. Shlens, and C. Szegedy. Explaining and Harnessing Adversarial Examples, Mar. 2015.

B. Lakshminarayanan, A. Pritzel, and C. Blundell. Simple and Scalable Predictive Uncertainty Estimation using Deep Ensembles. In *Advances in Neural Information Processing Systems*, volume 30. Curran Associates, Inc., 2017.
